# Investigation of the anti-Huanglongbing effects using antimicrobial lipopeptide and phytohormone complex powder prepared from *Bacillus amyloliquefaciens* MG-2 fermentation

**DOI:** 10.3389/fmicb.2024.1458051

**Published:** 2024-12-18

**Authors:** Zhicheng Ding, Yang Liu, Shaoran Zhang, Fangkui Wang, Qi Zong, Yuehua Yang, Anna Du, Yajie Zheng, Jian Zhu, Ling Jiang

**Affiliations:** ^1^National Key Laboratory of Germplasm Innovation and Utilization of Horticultural Crops, National Fruit Free-Virus Germplasm Resource Indoor Conservation Center, Department of Horticulture and Forestry, Huazhong Agricultural University, Wuhan, China; ^2^National Key Laboratory of Agricultural Microbiology, Wuhan, China

**Keywords:** lipopeptide, *Candidatus* Liberibacter asiaticus, inducing plant immunity, growth-promoting effect, microflora analysis, metabolite, phytohormone

## Abstract

Global citrus production has been severely affected by citrus Huanglongbing (HLB) disease, caused by Candidatus Liberibacter asiaticus (Clas), and the development of effective control methods are crucial. This study employed antimicrobial lipopeptide and phytohormone complex powder (L1) prepared from the fermentation broth of the endophytic plant growth promoting bacterium (PGPB) of *Bacillus amyloliquefaciens* strain MG-2 to treat *Candidatus* Liberibacter asiaticus (*C*Las)-infected ‘*Citrus reticulata* ‘Chun Jian’ plants. Real-time fluorescence quantitative polymerase chain reaction (qPCR) and PCR were employed for disease detection. The results revealed that after 15 spray-drench treatments with L1 solution, the HLB infection rate decreased from 100 to 50%, the bacterial titer decreased by 51.9% compared with a 27.9% decrease in the control group. L1 treatment triggered the production of reactive oxygen species, increased lignin content, and increased defense enzyme activities (*p* < 0.05). Defense-related gene expression significantly increased within 12 h of treatment. In addition, L1 application also promoted plant growth, as evidenced by higher transpiration rates and net photosynthetic rates as well as increased leave or root density. Root flora analysis revealed that the abundances of *Burkholderia_thailandensis*, *unclassified_g_Burkholderia-Caballeronia-Paraburkholderia*, *unclassified_g__Allorhizobium-Neorhizobium-Pararhizobium-Rhizobium*, and *Pseudomonas_mosselii* were 1.64, 1.46, 5.84, and 6.93 times greater, respectively, than those in the control group. The levels of phenylpropanoids, polyketides, lipids, lipid-like molecules, organic acids, and derivatives, significantly increased following L1 treatment (FC > 2, *p* < 0.05). Additionally, salicylic acid, dihydrojasmonic acid, and isopentenyl adenosine levels in leaves markedly increased. High-performance liquid chromatography (HPLC) confirmed that L1 contained surfactin, iturin and fengycin cyclic-lipopeptides (CLPs) as well as indole-3-acetic acid (IAA), 3-indolebutyric acid (IBA), *indole-3-carboxylic acid* and *indole-3-carboxaldehyde auxins*, *N6-*entopentenyladenine and t-zeatin-riboside cytokinins, abscisic acid, 1-aminocyclicpanecarboxylic acid, salicylic acid, and gibberellin A1, A3 and A4 phytohormones. These findings provide insight into multiple mechanisms by which endophytic Bacillus PGPB L1 is able to combat HLB disease, to promote citrus plant growth, and to optimize the root flora for soil health which offering an innovative strategy for sustainable management of this severe disease and improving citrus plant growth and productivity

## Highlights

- The lipopeptide and phytohormone complexes derived from the fermentation of *Bacillus amyloliquefaciens* MG-2 have demonstrated efficacy in reducing *C*las titers and stimulating host immune resistance.
- L1 treatment upregulated secondary metabolites and the contents of the hormones SA, H2JA and IPA in leaves, providing a foundation for combating HLB.
- Indole-3-acetic acid (IAA), 3-indolebutyric acid (IBA), *indole-3-carboxylic acid*, *indole-3-carboxaldehyde*, N6-isopentenyladenine, and gibberellin A1 contained in the L1 complex lay an important foundation for growth promotion.
- Following the L1 treatment, there was a significant increase in citrus growth, with increased root activity and greater photosynthetic efficiency. Activated ROS and defense enzyme activity.
- Flora analysis confirmed that, following L1 treatment, beneficial probiotics such as *Burkholderia thailandensis*, the unclassified genus *Burkholderia-Caballeronia-Paraburkholderia*, the unclassified genus *Allorhizobium-Neorhizobium-Pararhizobium-Rhizobium*, and *Pseudomonas mosselii* were enriched in the root system and beneficial to soil health.

## 1 Introduction

Citrus Huanglongbing (HLB) disease poses a major obstacle to citrus production globally (De Francesco et al., 2022). With climate change, the range of Asian citrus psyllid (ACP), which is responsible for transmitting HLB pathogens, has been steadily expanding in China (Wang R. et al., 2020; Sétamou et al., 2020). The pathogen known as *Candidatus* Liberibacter asiaticus (*C*Las) causes HLB-related disease (Jagoueix et al., 1994). Infected citrus leaves exhibit symptoms such as yellowing and mottling, whereas fruits experience stunted growth and deformities (Inoue et al., 2020). Prolonged infection with *C*Las can lead to root collapse and disruption of the root-associated microbial community. This disruption is characterized by changes in key taxa and an increase in soil-borne fungi such as *Fusarium and Phytophthora* (Ginnan et al., 2020). Cultivating *C*Las in its pure culture remains a challenge, and citrus varieties resistant to HLB disease are not yet available. Managing HLB disease in infected citrus plants is a complex task that greatly impedes the sustainable development of the citrus industry (Li J. et al., 2019). Several treatments such as antibiotic (Zhang et al., 2015), stable antimicrobial peptide (Huang et al., 2021), oligonucleotides, transgenic technology (Peng et al., 2021; Longhi et al., 2022), targeted small molecule inhibitors (Pagliai et al., 2014), and the use of *Bacillus* and Streptomyces species against HLB disease (Tang et al., 2018; Asad et al., 2021; Li J. et al., 2022) have been investigated. However, the search for a safe, effective and plant growth solutions for combating HLB disease and citrus productivity are still required.

Cyclic-lipopeptides (CLPs) produced by endophytic plant growth promoting bacteria (PGPB) have undergone extensive research because of their potent bactericidal or fungicidal activity. The CLPs can be classified into three major families: surfactins, iturins, and fengycins (Rahman et al., 2021; Assena et al., 2024). The CLP metabolites are produced from *Bacillus subtilis*, *amyloliquefacins or velezensis*, *fermentation* (Hamley, 2015). The surfactins are a cyclic compound composed of 7 *α*-amino acid residues linked to C13–15 *β*-hydroxy fatty acids. There are potent contact bactericides (Qiao et al., 2024). Recently, Surfactins or PGPB have been shown to induce systemic resistance (IRS) in plants. They stimulate plant immunity by activating the innate immune system and providing long-lasting disease protection (Yu et al., 2022; Zhu et al., 2022; Su et al., 2024). Iturin and fengycin are cyclic-lipopeptides consisting 7 α-amino acid residues connected by *β*-amino C14–17 and C14-18 fatty acids, respectively. They are more activity against various plant pathogenic fungi (Saggese et al., 2018). The antimicrobial activity and ISR protection of surfactins or PGPR are effective mechanisms for citrus *C*Las control.

Endophytic *Bacillus* PGPB not only reside within plants but also have significant physiological effects on their host plants. They promote plant or root growth with phytohormone metabolites and facilitate nitrogen fixation as well as nutrient use efficiency such as phosphate solubilization and abiotic stress tolerance (Das Mohapatra et al., 2024; Zahra et al., 2024; Marappa et al., 2020). Earlier *Bacillus velezensis* study demonstrated significant citrus plant growth and branching as well as high root mass by soil treatment of whole fermentation powder (Bai et al., 2024).

As the incidence of HLB disease progressed, the populations of five endophytic bacteria, namely, *Delftia*, *Acinetobacter*, *Pseudomonas*, *Burkholderia,* and Microbacterium, increased in navel orange leaves. Consequently, the study of the root microbiota in citrus health research has become a key area of focus in investigations of HLB infection (Zhang et al., 2013; Blaustein et al., 2017; Srivastava et al., 2022).

The aim of this study was to investigate the efficacy and citrus plant growth effect of cyclic-lipopeptide and phytohormone complex powder (L1) prepared from *Bacillus amyloliquefaciens* strain MG-2 fermentation in controlling ‘Chunjian’ citrus plants infected with *C*Las. Quantitative polymerase chain reaction (qPCR) and polymerase chain reaction (PCR) were used to monitor pathogen titers. Changes in reactive oxygen species levels, immune-related gene expression, defense enzyme activity, morphological indices of citrus plants, microbial community structure in roots, and non-target metabolites and endophytic hormones of leaves and roots were analyzed. These investigations aimed to elucidate the mechanisms underlying *C*Las resistance, citrus growth-promoting effects, and optimization of the root flora in L1 treated plants. The ultimate goal is to provide innovative and sustainable solutions for long-term control of *citrus green disease* and improving plant growth and abiotic stress tolerance for yield increase and fruit quality improvement.

## 2 Materials and methods

### 2.1 Plant materials and basic management

HLB-positive nursery plants were obtained via qPCR from a group of *C*Las-infected citrus nursery plants. *Citrus reticulata* ‘Chun Jian’ plants were cultivated in a greenhouse, with *Poncirus trifoliata* used as the rootstock. Daily management practices were followed, including the use of 1/8 MS (Murashige and Skoog, 1962) medium containing essential and trace elements at a dosage of 200 mL per plant. Additionally, a compound fertilizer with an N: P_2_O_5_:K_2_O ratio of 15:15:15 (Yichang Fusheng Chemical Co., LTD) was applied at a rate of 10 g/plant. Organic manure is fermented chicken manure (Shijiazhuang Xinwang breeding base), 10 g/plant. This fertilization regimen was repeated every 10 days for a total of five applications.

### 2.2 Methods for treating *C*Las-infected plants with lipopeptide and phytohormone complexes (L1)

The L1 powder used in this study was provided by Dr. Sheng Liu. The lipopeptide and phytohormone complex powder (L1) was prepared from *Bacillus amyloliquefaciens strain* MG-2 fermentation, and its genetic background was based on GenBank information (WER36268.1), which included the bacillomycin D hybrid PKS/nonribosomal peptide (NRPS) *Bam*A, consisting of polyketide synthase (PKS) and NRPS. 3,000 L fermentation medium: corn pulp powder 35 kg, sweet potato starch 40 kg, amino acid powder 30 kg, soybean meal powder 10 kg, glucose 10 kg, (NH4)_2_HPO_4_ 2.0 kg, MgSO_4_ 1.8 kg, KH_2_PO_4_ 3.2 kg, NaCl 6.0 kg, defoaming agent 2 L. The fermentation culture conditions were as follows: inoculation amount 5%, culture temperature 26–30°C, culture time 30 h, stirring speed 200 rpm. The fermentation solution is subjected to wall breaking treatment, the suspension is centrifuged, and the concentrated solution after centrifugation is filtered, methanol extraction is carried out, and then the concentrated solution of cyclic-lipopeptide and phytohormone can be obtained by demethanol treatment. The concentrated solution after centrifugation is spray-dried to obtain our cyclic-lipopeptide and phytohormone metabolites powder and mixed with 50% inert ingredients to obtained stable L1 powder formulation containing 50% fermentation solids. For the test, the L1 powder was diluted 500 times with water for the following experimental design: 600 mL/plant (100 mL leave spray- 500 mL soil drench) treatments; CK plants were treated with 600 mL/plant of water. From mid-June 2022 to the end of March 2023, the experiment was conducted once every 7 days, resulting in a total of 15 treatments and 18 treatments.

### 2.3 PCR and qPCR methods for the detection of citrus HLB disease

#### 2.3.1 DNA extraction

On the 105th day after L1 treatment, two leaves were collected in each direction from the tip of a branch in four directions: east, south, west, and north. The citrus leaf veins were carefully clipped and immediately placed in centrifuge tubes containing an ice bath. To preserve the samples for future use, liquid nitrogen treatment was applied, and the samples were stored at −80°C. The genomic DNA from the citrus leaf veins of 100 mg/sample was then extracted via the CTAB method following the instructions of the plant genome DNA extraction kit (No. ZP309-30, ZOMANBIO Co., Ltd.). The sampling method of other time points is the same as above.

#### 2.3.2 Detection of citrus HLB disease pathogens via PCR

PCR amplification was performed with the OI1/OI2c primer pair (Jagoueix et al., 1994). The amplification procedure included denaturation at 94°C for 3 min, followed by denaturation at 94°C for 30 s, annealing at 62°C for 30 s, and extension at 72°C for 1 min, resulting in 35 cycles. A final extension step was performed at 72°C for 10 min. Sterilized distilled water was used as the negative control, whereas plasmids with the target fragment of the Huanglong disease pathogen from our laboratory served as the positive control. The PCR kit instructions from CWBIO Reagent Biotechnology Co., Ltd. (Beijing) were followed, and three biological duplications were performed.

#### 2.3.3 Establishment of standard curves and qPCR detection methods

DNA preparations were adjusted to 100 ng/μl and stored at −20°C. The qPCR analysis was conducted via a Corbett RG 6000 (Germany) fluorescent quantitative PCR instrument and the Sybr Green qPCR kit produced by CWBIO Reagent Biotechnology Co., Ltd. (Beijing). Each DNA sample subjected to qPCR was analyzed, three biological replicates. The primer information can be found in Supplementary Table S1. The target gene studied was A04, with *COX* serving as the housekeeping gene (Li et al., 2006; Hu et al., 2006). The detection procedure was validated via a standard curve in which a fragment of *A04* was used as a target gene, ‘ccaacgaaaagatcagatattcctaatactagtatcacggatagcaatcttgacgagacgatttttggcaacttttacacctcca’, was obtained from the following gene number: CP001677.5. In addition, the house-keeping gene sequence of *COX* used to construct a plasmid vector, ‘gtatgccacgtcgcattccagattatccagatgcttacgctggatggaatgcccttagcagttttggc’, was obtained from the following gene number: CX297817.1. The work used to construct the standard curve for HLB disease detection is shown in Supplementary Figures S1A–D. In the qPCR steps for the HLB disease pathogen, denaturation was performed at 94°C for 3 min, followed by denaturation at 94°C for 5 s, annealing at 56°C for 30 s, and extension at 72°C for 35 s. A total of 35 cycles were carried out, with a final extension step at 72°C for 10 min. Each sample included three biological replicates. The DNA extracted from the leaf veins of ‘*Citrus reticulata Banco*’ plants that tested positive in the laboratory was used as the positive control for qPCR, whereas the callus DNA of “*Citrus sinensis’valencia’* was used as the negative control. The analysis of test data was performed via the 2^-ΔΔCT^ method (Livak and Schmittgen, 2001).

### 2.4 Method for detecting morphological and physiological indices

#### 2.4.1 Plant morphological indices

After 15 treatments, the root projection area was calculated by selecting roots via the histogram option of Photoshop 2018 software. The leaf area was computed via the following formula: number of leaves × length × width × (2/3), as specified by Hu (2015). Each group consisted of 6 biological replicates.

Test method of I-KI_2,_ was performed, 0.3 g of iodine and 1.3 g of potassium iodide were weighed, added to a small beaker with approximately 99 mL of distilled water, and then transferred to a 100 mL volumetric bottle. The I-KI_2_ solution was used for section staining. The leaf veins were sandwiched between two potato slices and cut several times with a razor blade to obtain extremely thin cross-cut slices of the leaf veins. A small amount of distilled water was added to the Petri dish to flatten the slices, which were then placed on a slide using small tweezers. The slices were observed under a microscope, and thin slices were selected for immediate focus imaging. Using a graduated pipette, 0.3 mL of I-KI_2_ solution was added quantitatively, and the samples were stained for 2 min. A thin section was then selected, focused, and photographed immediately. The eyepiece was set to 10x and the objective to 30x.

#### 2.4.2 Determination of physiology indices

##### 2.4.2.1 Photosynthetic rate

The net photosynthetic rate, transpiration rate, intercellular carbon dioxide concentration, and stomatal conductance of the plants were measured via a portable photosynthetic apparatus called Li-6800 (Li-COR, Lincoln, USA). The measurements were conducted in a well-lit, well-ventilated area in the morning of mid-October 2022. Mature leaves located near the middle of the branch were selected, and three readings were taken from different directions for each plant. The average value was then calculated (Wang T. et al., 2020).

##### 2.4.2.2 Chlorophyll content

The SPAD value and nitrogen content were determined via a TYS-4 N chlorophyll analyzer (Zhejiang Topu Yunnong Technology Co., Ltd.).

##### 2.4.2.3 Starch content

The starch content was measured via a starch content kit following the procedures outlined by Beijing Solaibao Technology Co., Ltd. (Zhang et al., 2020).

##### 2.4.2.4 Lignin content

The lignin ELISA kit was obtained from Jiangsu Enzyme Free Industry Co., Ltd. The lignin content in the samples was determined via a double-antibody sandwich method (Srivastava et al., 2015).

For the determination of the above four indicators, six biological replicates were included for both the L1 treatment and the control groups. The test data were analyzed via Excel 2021 software and SPSS 22.0 software.

#### 2.4.3 Staining detection of reactive oxygen species content

A DAB (3,3′-diaminobenzidine tetrahydrochloride) staining kit, purchased from Nanjing Jiancheng Bioengineering Research Institute Co., Ltd., was used for the L1 treatment groups and the control group during the 15th treatment for a duration of 8 h. Tissue staining was carried out promptly upon sample collection following the specific method described in the literature (Daudi and O'Brien, 2012).

Additionally, the BCIP/NBT (5-bromo-4-chloro-3-indolyl phosphate) alkaline phosphatase color-developing kit was acquired from Nanjing Jiancheng Bioengineering Research Institute Co., Ltd. The specific method described in the literature was followed (Jérôme et al., 2019).

#### 2.4.4 Method for detecting defense enzyme activity and pH

##### 2.4.4.1 Enzyme activity in leaves

The determination of enzyme activity in leaves involves various principles. Polyphenol oxidase (PPO) catalyzes the production of quinone from the substrate phenol, which is characterized by light absorption at 420 nm (Zhu et al., 2019). The activity of PPO can be measured by monitoring the change in absorbance at 420 nm. Peroxidase (POD) catalyzes the oxidation of polyphenolic aromatic substances in the presence of hydrogen peroxide, and POD activity can also be determined by measuring the change in absorbance at 420 nm (Li et al., 2013). Phenylalanine ammonia-lyase (PAL) breaks down L-phenylalanine into trans-cinnamic acid and ammonia. The PAL activity was calculated by monitoring the change in the absorption value at 290 nm (Kostenyuk et al., 2002). Superoxide dismutase (SOD) activity was determined via an enzyme-linked meter. The interaction of the superoxide anion (O^−^) with water-soluble tetrazolium-1 (WST-1) was measured at its maximum absorption peak at 450 nm (Peskin and Winterbourn, 2017). Catalase (CAT) activity is determined by measuring the change in absorbance at 405 nm after CAT reacts with decomposing hydrogen peroxide (H_2_O_2_) and ammonium molybdate (Zhang et al., 2008). All kits used for these determinations were procured from Nanjing Jiengcheng Bioengineering Research Institute Co., Ltd. Six biological replicates were established for each experimental group, and statistical analysis was performed via Excel 2021 and SPSS 25.0 software to conduct variance and significance level tests.

##### 2.4.4.2 Enzyme activity in the soil

To determine soil phenoloxidase activity, 0.1 g of air-dried soil with a particle diameter less than 2 mm was placed into a 2 mL centrifuge tube following the methodology outlined in the literature (Jabborova et al., 2021). The analysis was carried out according to the procedure described in another reference (Myszura et al., 2021).

##### 2.4.4.3 Determination of the soil pH

Soil samples from the topsoil (0–20 cm) were collected randomly from different points in the citrus cultivation area, 10–30 cm away from the citrus trunk, and mixed thoroughly. A 10.00 g soil sample was weighed into a 50 mL tall beaker or a suitable container and mixed with 25 mL of water. pH was measured following the instructions provided by the manufacturer.

### 2.5 Determination of the relative expression of plant resistance-related genes

To determine the relative expression of plant resistance genes, target gene sequences related to resistance were searched in the NCBI database. The primers used were designed via Primer3 (Supplementary Table S2).

#### 2.5.1 Sampling method

24 h after the 15th treatment, samples were collected from the CK and L1 treatment groups. Four to six leaves were randomly taken from branches in the east, south, west, and north directions and then cut and divided into three samples for each plant.

#### 2.5.2 Extraction of RNA

A 50 mg leaf sample was ground in liquid nitrogen, and 1 mL of RNAiso Plus (TAKARA) reagent was added to each 50 mg of tissue. The tissue was homogenized via a homogenizer according to the instructions provided with the kit.

#### 2.5.3 Synthesis of cDNA

A reverse transcription kit was purchased from CWBIO Reagent Biotechnology Co., Ltd. The RNA was incubated at 45°C for 25 min and then inactivated at 85°C for 5 min. The resulting cDNA was stored at −20°C.

#### 2.5.4 qPCR

The cDNA was diluted to 200 ng/μl and used as a template for qPCR, with three biological replicates per treatment group. The six target genes (*nho1, GST1, HSP90, WRKY22, WRKY24*, and *WRKY33*) were analyzed, with Actin serving as the housekeeping gene. The qPCR involved denaturation at 94°C for 3 min, followed by 40 cycles of denaturation at 94°C for 5 s, annealing at 56°C for 35 s, extension at 72°C for 35 s, and a final extension step at 72°C for 10 min. Quantitative results were analyzed via the relative quantitative 2^-ΔΔCT^ method (Livak and Schmittgen, 2001).

### 2.6 Microflora analysis methods

#### 2.6.1 Sample collection

The L1 treatment group and control groups each had four biological replicates. After 15 treatments, the root samples were randomly selected and stored at −70°C in a refrigerator for subsequent high-throughput sequencing analysis.

#### 2.6.2 Illumina high-throughput sequencing and dilution curve analysis

Genomic DNA was extracted from the root samples and subjected to 1% agarose gel electrophoresis. The V3-V4 region primer (799F_1193R) was used for amplification of the 16S rDNA of bacteria. The Illumina-sequenced PE reads were initially spliced on the basis of overlapping regions, and then the sequences were subjected to quality control and filtering. After sample differentiation, operational taxonomic unit (OTU) cluster analysis and species taxonomic analysis were performed. The significance of differences between various groups was determined via the Wilcoxon rank-sum test at the OTU level.

#### 2.6.3 Software and database for biological information analysis

Paired-end sequences were assembled via the Flash 1.2.11 analysis database to produce a taxonomic abundance table. Beta diversity distance was measured via QIIME 1.9.1, Uparse 11 was used for OTU clustering, alpha diversity analysis was performed with Mothur 1.30.2, and Fastp 0.19 was used for quality control analysis.

### 2.7 Nontargeted metabolite analysis methods

#### 2.7.1 Sample extraction method

For each citrus plant that underwent 15 treatments with L1, leaves and roots were collected from various directions, with six biological replicates each. The samples were immediately frozen in liquid nitrogen and stored at −70°C. The samples were subsequently treated with a precooled methanol/acetonitrile/aqueous mixture (2:2:1, v/v), followed by vortex mixing, ultrasonic treatment, and centrifugation at 14000 × g for 20 min at 4°C. The dried extract was then dissolved by adding a solution containing acetonitrile and water at a 1:1 (v/v) ratio. After the mixture was swirled, it was centrifuged again at 14000 × g for an additional 15 min at 4°C, and the supernatant was collected for further analysis.

#### 2.7.2 Chromatography–mass spectrometry analysis

The samples were separated via an Agilent 1,290 Infinity LC ultrahigh-performance liquid chromatography (UHPLC) C-18 column (Ivanisevic et al., 2013). Q-TOF mass spectrometry was performed using an AB Triple TOF 6600 mass spectrometer to acquire primary and secondary spectra of the samples.

#### 2.7.3 Data analysis

The original data in Wiff format were converted to mzXML format via ProteoWizard. Subsequently, peak alignment, retention time correction, and peak area extraction were performed via MSDAIL software. The metabolome of citrus samples was investigated via an in-house database (Shanghai Applied Protein Technology) as a reference (Luo et al., 2017; Gu et al., 2018).

### 2.8 Methods for determining phytohormones

#### 2.8.1 Sample extraction

Weighing 0.1 g of samples into mass spectrometry water, Liquid nitrogen grinding samples. Take sample of 100 μL and add 400 μL precipitator (acetonitrile: Water =1:1), vortex mixing, extraction at 4 ° C for 30 min, centrifuge at 12000 rpm4°Cfor 10 min, take 300 μL supernatant slowly through the extraction column, add 500 μL eluent (30% acetonitrile), slowly through the extraction column, and mix the two times of flow solution, LC–MS analysis. There were 4 biological replicates per treatment.

#### 2.8.2 Standard solution preparation

The stock solution of individual phytohormone was mixed and prepared in phytohormone-free matrix to obtain a series of phytohormone calibrators. Certain concentrations of Indole-3-acetic acid-D4, Jasmonic acid-D5, N6-Isopentenyladenine-D6, Dihydrozeatin-D3, Gibberellin A1-D4, Salicylic acid-D4 and Abscisic acid-D6 were mixed as Internal Standard (IS). The stock solution of all of these and working solution were stored in refrigerator of −20°C.

#### 2.8.3 LC–MS method

LC–MS method phytohormone content determination was conducted according to the instructions of the Clinical and Laboratory Standards Institute (CLSI). An ultrahigh-performance liquid chromatography coupled with tandem mass spectrometry (UHPLC–MS/MS) system (ExionLC™ AD UHPLC-QTRAP® 6,500+, AB SCIEX Corp., Boston, MA, USA) was used. The liquid chromatography–mass spectrometry methods used were based on the approved guideline CLSI document C62-A (Wayne, PA: Clinical and Laboratory Standards Institute, 2014), and were performed at Novogene Biotechnology Co., Ltd. Chromatographic column: Waters XSelect HSS T3 (2.1 × 150 mm, 2.5 μm), Mobile phase: Phase A: 0.01% formic acid water phase B: 0.01% formic acid acetonitrile, Column temperature: 45°C, Sample size: 10 μL, Flow rate: 0.3 mL/min. All of the phytohormone standards and stable isotope-labeled standards were obtained from ZZ Standards Co., LTD. (Shanghai, China). Methanol (Optima LC–MS), acetonitrile and formic acid were purchased from Thermo-Fisher Scientific (FairLawn, NJ, USA). Ultrapure water was purchased from Millipore (MA, USA). Separation was performed on a Waters XSelect HSS T3 column (2.1 × 150 mm, 2.5 μm) which was maintained at 45°C. The mobile phase, consisting of 0.01% formic acid in water (solvent A) and 0.01% formic acid in acetonitrile (solvent B), was delivered at a flow rate of 0.30 mL/min. The solvent gradient was set as follows: initial 10% B, 1 min; 10–50% B, 3 min; 50–65% B, 4 min; 65–70% B, 6 min; 70–100% B, 7 min; 100–10% B, 9.1 min; 10% B, 12 min.

### 2.9 Methods for determining lipopeptides

#### 2.9.1 Determination of iturin A

The dry powder of L1 complex was weighed at 0.05 g and dissolved in 1 mL methanol solution. The clarified supernatant was obtained by centrifugation at 4°C and 12,000 r/min for 5 min. Which was filtered by 0.22 μm polyacrylamide organic filter membrane and then used for HPLC analysis and identification. The HPLC analysis system was SHIMADZU LC20A, the chromatographic column was Agilent TC-C18, 5 μm, 4.6 × 250 mm, and the following three lipids were used in this system. Iturin A HPLC assay was performed with the detection wavelength at 210 nm and column temperature at 30°C. The samples were analyzed by gradient elution with 10 mM methanol as mobile phase A and pure acetonitrile as mobile phase B. The ratio of iturin A mobile phase A:B = 65:35 (V/V). A: 60–93%, B: 40–7%; Flow time 12 min to A: 93%, B:7%. The flow rate was 1.0 mL/min and the sample size was 10 μL.

#### 2.9.2 Determination of Surfactin

Weigh 0.1 g of the dry powder of L1 complex and dissolve it in 1 mL methanol solution. The preparation method of supernatant is the same as above. Surfactin was analyzed by HPLC at a wavelength of 205 nm, a column temperature of 30°C, and a flow time of 20 min. Samples were analyzed using pure methanol as blank control and equal gradient elution. Mobile phase A was 10 mM acetonitrile, mobile phase B was pure methanol, and the ratio of iturin A mobile phase A:B = 60:40 (V/V). A: 60–93%, B: 40–7%; 10 min to A: 93%, B:7%. The flow rate was 1.0 mL/min. The sample size was 10 μL. Flow time 20 min.

#### 2.9.3 Determination of Fengycin

Weigh 0.1 g of the dry powder of L1 complex and dissolve it in 1 mL methanol solution. The preparation method of the supernatant was the same as that of Iturin A. The detection wavelength was 210 nm, the column temperature was 30°C, and the flow time was 20 min. Chromatographic methanol was used as blank control, and gradient elution was used for sample analysis. Mobile phase A was chromatographic grade pure water, mobile phase B was chromatographic pure acetonitrile, and the ratio of Fengycin mobile phase A:B = 70:30 (V/V). A: 70–93%, B: 30–7%; 10 min, A: 93%, B:7%. The flow rate was 1.0 mL/min. The sample size was 10 μL. Flow time 15 min.

The concentration gradients were designed with the standard samples of three kinds of lipopeptides (Shanghai Yuanye Biotechnology Co., LTD), and the standard curves were prepared with above mentioned procedure, respectively.

## 3 Results

### 3.1 Anti-HLB effects and the induced immune response of L1 powder

Standard curve analysis of the results of the qPCR detection of HLB disease was conducted. The results are presented in Supplementary Figures S2A,B, whereas amplification curves are shown in Supplementary Figures S2C,D, and melting curves are displayed in Supplementary Figures S2E,F. The primer efficiency of COX+/COX-, which targets the citrus internal reference gene, was determined to be 102.49%, with a minimum detection limit of 112.65 copies. For the A04+/A04- primers specific for HLB disease, the primer efficiency was 91.88%, with the lowest detection limit of 230.98 copies. Under the instrumental conditions of this laboratory, the use of the above primers for *C*las detection could achieve high sensitivity.

After 15 rounds of spray-drenching with L1, on the 15 times (105th) day posttreatment, the percentage of HLB-positive bacteria decreased from 6/6 initially to 3/6, on the 18 times posttreatment, the percentage of HLB-positive bacteria decreased from 6/6 initially to 1/6, indicating that L1 had a certain effect on reducing the *C*las titer. The percentage of positive cells in the control sample ranged from 6/6 to 5/6 (Table 1), potentially influenced by temperature and humidity changes (Supplementary Figure S3). Gel electrophoresis was used for PCR detection (Supplementary Figure S4). The raw qPCR data are available in the Supplementary Tables S3–S5. According to the standard curve equation, *Y* = -3.5333x + 39.451 (*R*^2^ = 0.9958), the bacterial titer (cells/gram tissue) in the L1-treated and control samples was calculated on the basis of CT cycle data. After L1 treatment for 15 times, the bacterial titer in the leaf vein decreased by 51.9%, while that in the control plant decreased by 27.9%, respectively. After a hypothesis test comparing the percentages, the calculated value of *u* = −17.686 and the test table value of *u*_0.01_ = 2.5758 indicated that the difference reached a very significant level. After L1 treatment for 18 times, the bacterial titer in the leaf vein decreased by 57.2%, while that in the control plant decreased by 31.9%, *u* = −18.37, and the test table value of *u*_0.01_ = 2.5758 indicated that the difference reached a very significant level (Supplementary Figure S5 and Supplementary Table S6). Although the titer also decreased in the control samples, the reduction in titer in the L1 treatment group was significantly greater than that in the control group.

**Table 1:** Detection results of HLB disease before and after treatment with antimicrobial lipopeptide in *Citrus reticulata* ‘Chun Jian’.

Processing and numbering	Before treatment 12-Nov-21			After 15 times treatment 22-Sep-22			After 18 times treatment 1-Mar-23	
Treatment L1	PCR	QPCR 2^-ΔΔCT^	Comprehensive judgment of HLB	PCR	QPCR 2^-ΔΔCT^	Comprehensive judgment of HLB	QPCR 2^-ΔΔCT^	Comprehensive judgment of HLB
C24	+	2.92 ± 0.38	+	−	0.91 ± 0.05	−	0.93 ± 0.06	−
C32	+	3.21 ± 0.26	+	+	1.51 ± 0.46	+	0.87 ± 0.22	−
C46	+	2.89 ± 0.87	+	−	0.86 ± 0.17	−	0.92 ± 0.13	−
C51	+	2.07 ± 0.25	+	+	1.69 ± 0.48	+	1.27 ± 0.38	+
C76	+	2.73 ± 0.41	+	+	1.13 ± 0.45	+	0.82 ± 0.11	−
C90	+	4.50 ± 0.71	+	−	0.66 ± 0.24	−	0.55 ± 0.27	−
Summary	Positive rate 6/6			Positive rate 3/6			Positive rate 1/6	
CK								
C4	+	3.98 ± 0.33	+	+	2.12 ± 0.11	+	2.18 ± 0.17	+
C15	+	2.09 ± 0.16	+	−	0.80 ± 0.19	−	0.77 ± 0.22	−
C17	+	1.78 ± 0.57	+	+	1.40 ± 0.15	+	1.35 ± 0.14	+
C48	+	2.10 ± 0.31	+	−	1.23 ± 0.85	+	1.20 ± 0.67	+
C70	+	2.91 ± 0.58	+	+	1.39 ± 0.53	+	1.45 ± 0.42	+
C97	+	5.52 ± 0.43	+	−	2.02 ± 0.92	+	2.14 ± 0.58	+
Summary	Positive rate 6/6			Positive rate 5/6			Positive rate 5/6	

Formula: ΔΔCT = (CT target gene -CT housekeep gene) x- (CT target gene -CT housekeep gene) ck.

X for treatment, o for control. 2^-ΔΔCT^ is the relative expression of the target gene, which is used to judge whether the sample carries Clas, 2^-ΔΔCT^ ≤ 1, the sample is negative, 2^-ΔΔCT^ > 1, the sample was positive.

#### 3.1.1 L1 can stimulate the production of ROS

**Figure 1:**
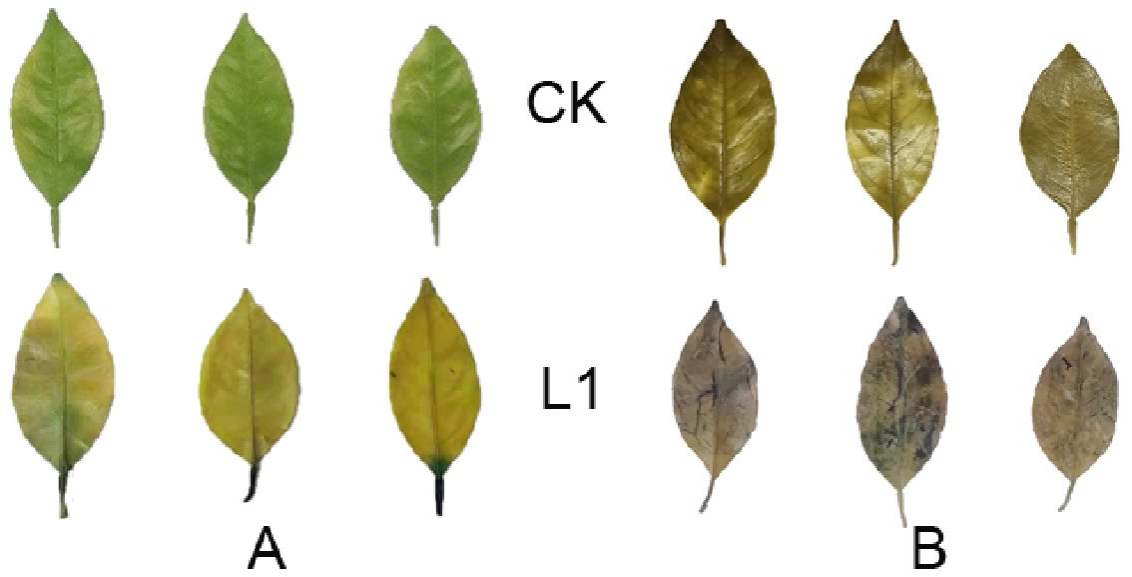
**(A,B)** Production of reactive oxygen species (ROS). **(A)** DAB staining; **(B)** NBT staining.

In this experiment, DAB staining was performed on citrus leaves *in vitro* after 15 repetitions of L1 treatment. Compared with those in the control group, the plants in the treatment group presented noticeable brown staining, indicating that L1 increased the accumulation of H_2_O_2_ and stimulated the immune response (Figure 1A). Additionally, NBT staining revealed prominent blue spots in the L1 treatment group, indicating that the application of L1 triggered the generation of superoxide anion radicals (O^2−^) and activated the oxidative stress response in ‘Chun Jian’ (Figure 1B).

#### 3.1.2 Changes in plant immune-related gene expression

**Figure 2:**
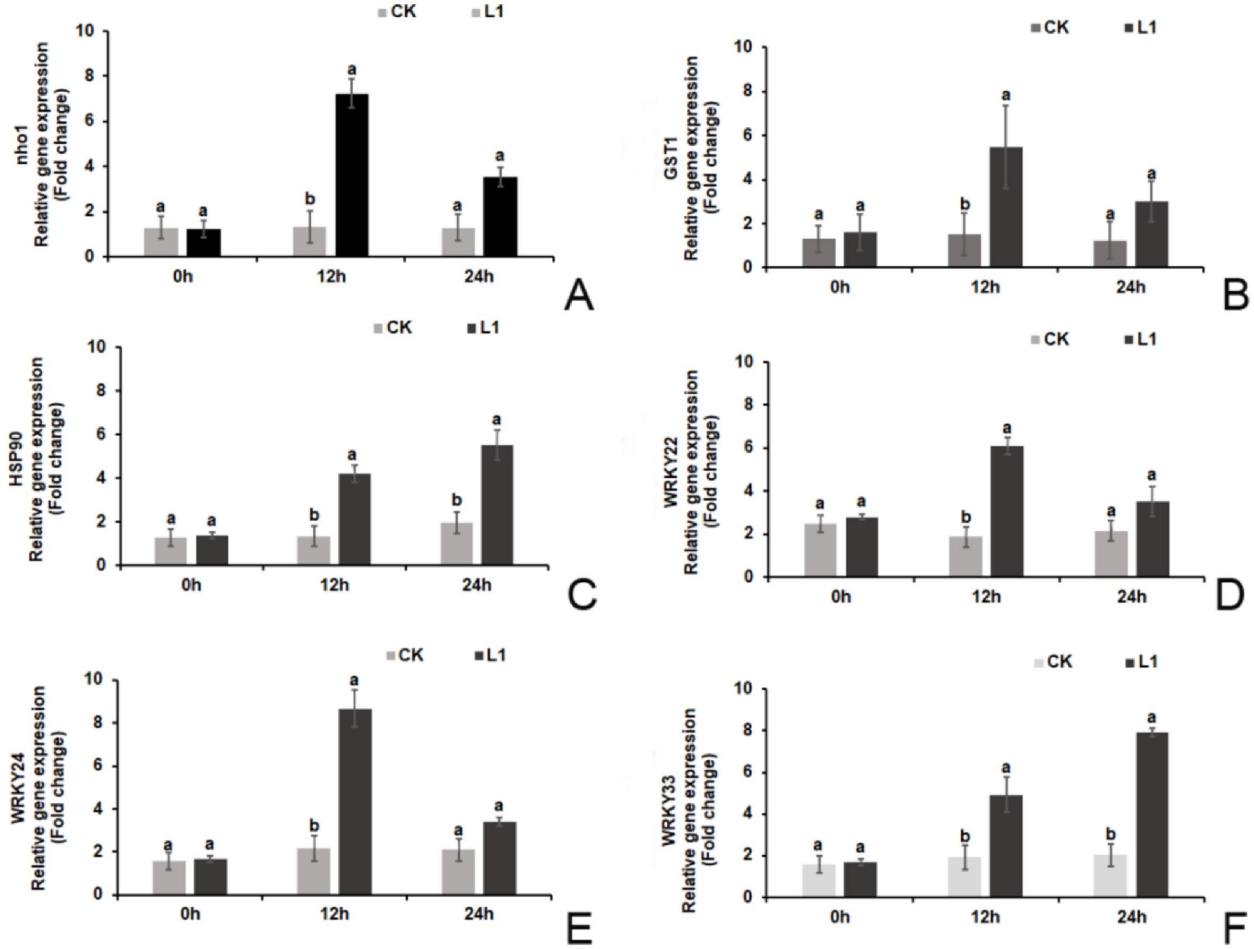
**(A–F)** Relative expression levels of plant immune-related genes. **(A)** nho1, **(B)** GST1, **(C)** HSP90, **(D)** WRKY22, **(E)** WRKY24, **(F)** WRKY33.

Prior to treatment (0 h), there were no significant differences in the relative expression levels of defense-related genes (*nho1*, *WRKY22, WRKY24, and WRKY33*) or hypersensitivity response genes (*GST1* and *HSP90*) between the L1 treatment group and the control group. However, at 12 h after treatment, the relative expression levels of these genes were significantly greater in the L1 treatment group than in the control group. Specifically, the relative expression levels of the 6 genes increased by 5.47-fold, 3.63-fold, 3.12-fold, 3.24-fold, 3.98-fold, and 2.57-fold, respectively. At 24 h after treatment, only the relative expression levels of *HSP90* and *WRKY33* were significantly greater in the L1 treatment group (2.82-fold and 3.89-fold, respectively). The relative expression levels of the other 4 genes were not significantly different from those in the control group (*p* < 0.05; Figures 2A–F).

#### 3.1.3 Changes in lignin content and defense enzyme

**Figure 3:**
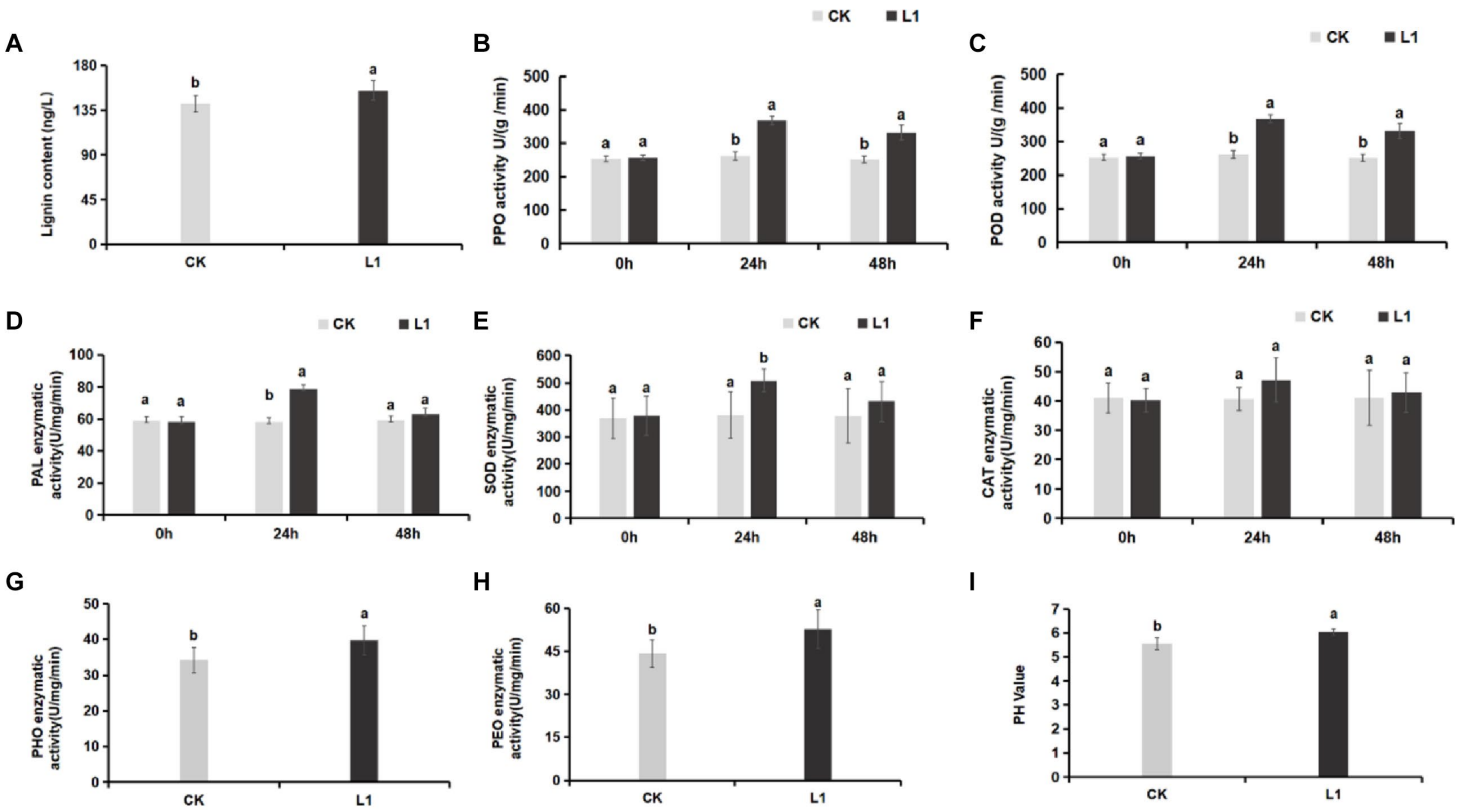
**(A–I)** Effects of L1 treatment on defense enzymes and soil pH. **(A)** Lignin, **(B)** PPO, **(C)** POD, **(D)** PAL, **(E)** SOD, **(F)** CAT, **(G)** PHO activity in soil, **(H)** PEO activity in soil, **(I)** soil pH. Different lowercase letters indicate significant differences (*p* < 0.05).

Lignin is an essential component of the plant cell wall, providing stability and resistance to external factors for normal plant growth and development. The results revealed that the lignin content in the control group was 141.84 ng/L, whereas in the L1 treatment group, it was 155.04 ng/L. The total lignin content in the L1 treatment group increased significantly by 9.31% (*p* < 0.05; Figure 3A).

Compared with those of the control, the activities of the defense enzymes PPO, POD, PAL, and SOD increased significantly, by 1.39-fold, 1.41-fold, 1.37-fold, and 1.33-fold, respectively, after 24 h of treatment with L1 (*p* < 0.05). Furthermore, the activities of the PPO and POD enzymes were notably greater in the treatment group than in the control group (*p* < 0.05) after 48 h of treatment with the antibacterial lipopeptide, with increases of 1.34-fold and 1.32-fold, respectively (Figures 3B–D). PPO primarily participates in the antioxidant defense system of plants, POD is involved in plant defense mechanisms, and PAL contributes to disease resistance and defense mechanisms in plants. SOD plays a crucial role as an antioxidant enzyme in plant cells and is responsible for eliminating O^2−^ within these cells. Additionally, there was no significant change in CAT activity (Figures 3E,F). These results indicate that L1 significantly enhances the function of these top 5 defense enzymes.

#### 3.1.4 Changes in peroxidase and phenoloxidase in soil

Soil peroxidase (PEO) is an enzyme present in the soil that facilitates the reduction of superoxide ions (O^2−^) and H_2_O_2_. Soil phenoloxidase (PHO) is an essential enzyme in plants, microorganisms, and other biological systems that aids in the absorption of nutrients in plants. It helps convert organic substances into nutrients such as NO^3−^ and PO_4_^3−^. Following treatment with the lipopeptide, the peroxidase and phenoloxidase levels in the soil increased by 15.33 and 19.29%, respectively, which were significantly greater than those in the control (*p* < 0.05; Figures 3G,H). Moreover, the pH of the soil increased from 5.56 to 6.05 (Figure 3I).

### 3.2 Growth-promoting effect of L1 powder

#### 3.2.1 Growth-promoting

**Figure 4:**
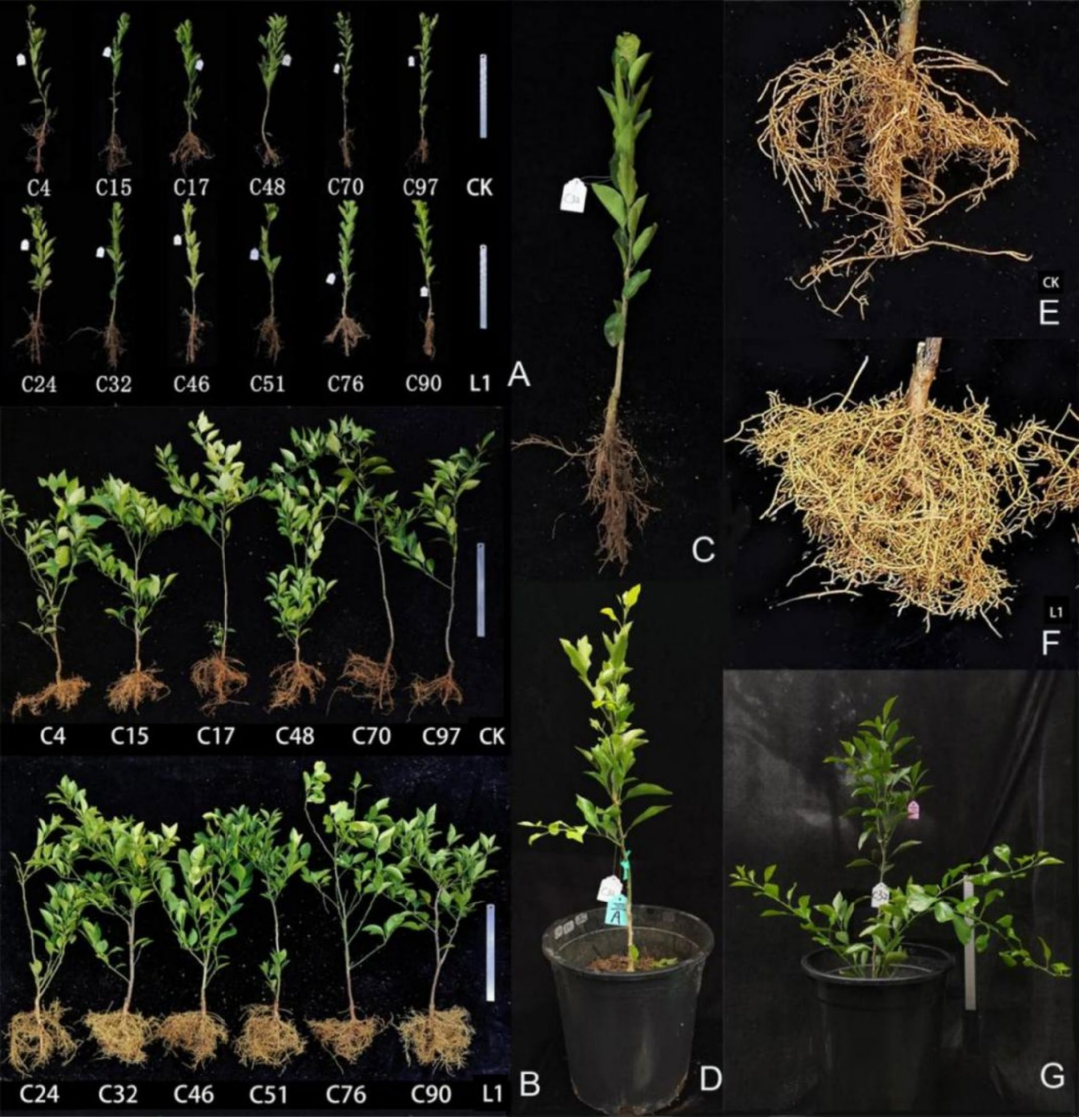
Morphological changes in *Citrus reticulata* ‘Chun Jian’ citrus nursery plants before and after treatment with L1. **(A)** After HLB detection, the positive plants were used for colonization on December 20, 2021; **(B)** After treatment 15 times from June to October 2022; **(C)** The image magnification of C32 in January 2022; **(D)** C32 in March 2022, before treatment, leaf yellowing at the branch tip in C32; **(G)** After treatment 15 times by L1, the leaves of C32 turned green on September 2022. **(E)** Root enlargement photograph of the CK plant; the roots browned. **(F)** Root enlargement photograph of the L1 treatment sample; the roots had developed well, and new roots were abundant. More vigor plant and branching as shown in L1 treated plants.

**Figure 5:**
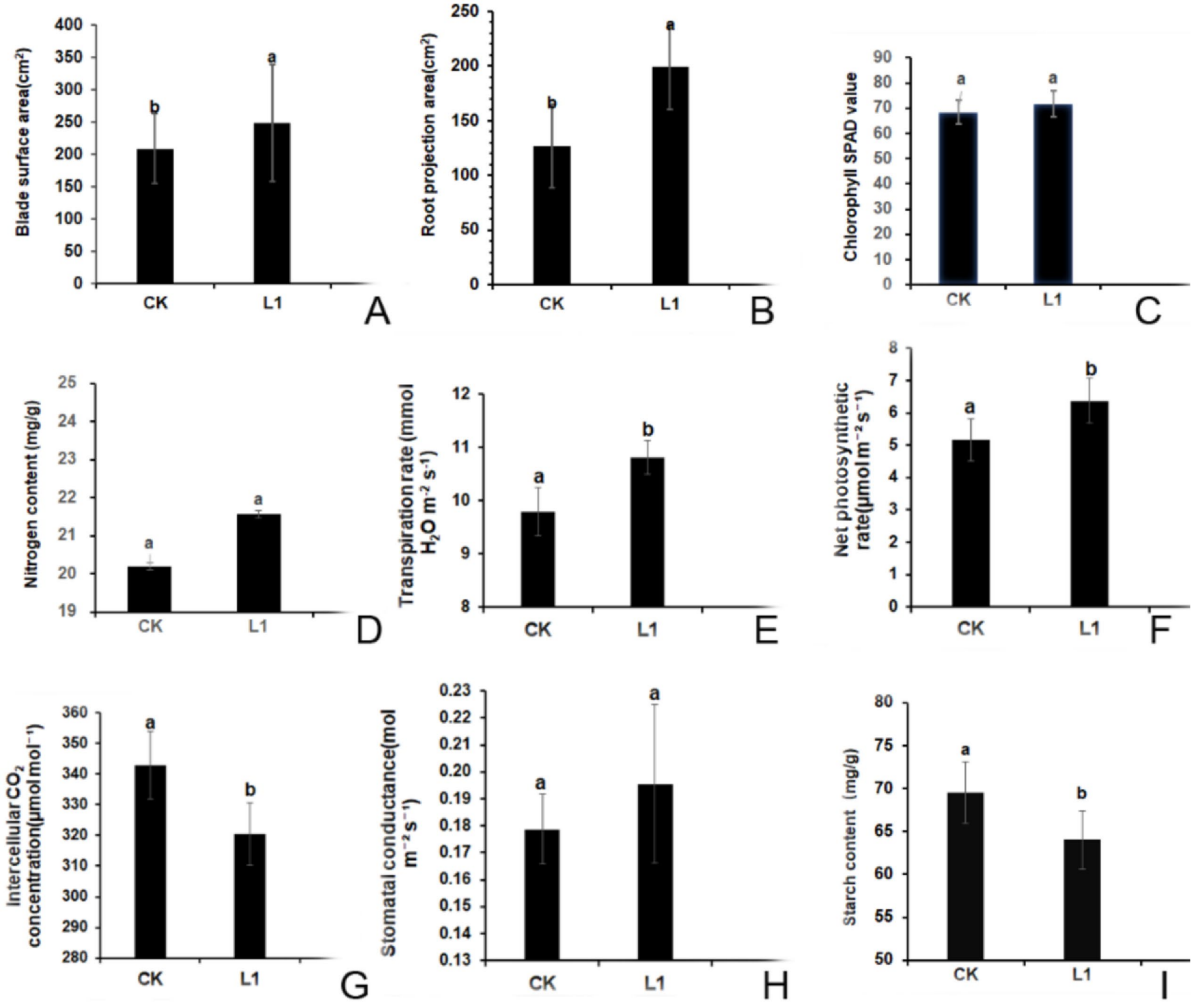
**(A–I)** Effects of L1 on the morphological and physiological indices of citrus plants. **(A)** surface area of the blade; **(B)** projected area of the root; **(C)** SPAD value; **(D)** nitrogen content; **(E)** transpiration rate; **(F)** net photosynthetic rate; **(G)** intercellular CO_2_ concentration; **(H)** stomatal conductance; **(I)** starch content. L1 was the treatment group, and CK was the control group. Different lowercase letters indicate that the difference reached a significant level (*p* < 0.05).

Growth-promoting effects Prior to treatment, the growth status of the test plants remained relatively stable (Figure 4A). After 15 L1 treatments, both the leaf area and the root projection area were significantly greater than those in the control group (Figures 5A,B). Compared with the control plants, the L1-treated plants presented lush growth and well-developed roots, indicating much better overall health (Figures 4B,F), while the roots appeared sparse and severely brown in control samples, as shown in the images (Figures 4B,E). Additionally, Figure 4C shows the enlarged image of the planted seedlings (Figure 4C), after 15 treatments, the top leaves of the L1-treated plants changed from yellow to green (Figures 4D,G).

#### 3.2.2 SPAD value and nitrogen content

After treatment with L1, the SPAD value increased by 4.9%, whereas the nitrogen content increased by 6.5% compared with that of the control group. However, these differences were not deemed significant according to Figures 5C,D.

#### 3.2.3 Photosynthesis indices

The results showed that, the transpiration rate, net photosynthetic rate, intercellular CO_2_ concentration and stomatal conductance in the control group were 9.79 mmol H_2_O m^−2^ s^−1^, 5.17 μmol m^−2^ s^−1^, 342.93 μmol mol^−1^, and 0.179 mol m^−2^ s^−1^, respectively. Compared with those in the control group, in L1 treatment group, the transpiration rate increased by 1.02 mmol H_2_O m^−2^ s^−1^ (Figure 5E), Pn increased by 1.21 μmol m^−2^ s^−1^ (Figure 5F), the intercellular CO_2_ concentration decreased by 22.50 μmol mol^−1^, and these three differences reached significant differences (Figure 5G), the stomatal conductance increased by 0.016 mol m^−2^ s^−1^, however, the difference was not significant (Figure 5H). This shows that the vital role of carbon dioxide was as a key component of photosynthesis, a lower intercellular concentration facilitates increased carbon dioxide involvement in photosynthetic reactions, consequentially boosting the net photosynthetic rate and, subsequently, the transpiration rate.

#### 3.2.4 Starch context

Following 15 rounds of treatment with antimicrobial lipopeptide, the starch content in the control group was 69.54 mg/g. In contrast, the L1 treatment group presented a significant reduction, with a starch content of 64.02 mg/g, representing a 7.94% decrease compared with that of the control group (*p* < 0.05; Figure 5I). These results indicate the effective mitigation of starch blockage in transfusion tissue induced by HLB disease through L1 treatment.

#### 3.2.5 I-KI_2_ staining test

The result indicated that after three-and-a-half months of treatment with L1, the leaves of *‘Citrus reticulata* ‘Chun Jian” were green, and the transverse sections of the veins were showed in Supplementary Figures S6A–C. The transverse segment of the vein is stained with I-KI_2_, and the sieve tubes and xylem tubes of the phloem showed only light blue in Supplementary Figures S6D–F.

The control samples showed the symptom of yellow-mottled, Supplementary Figures S6G–I showed the samples without staining. Supplementary Figures S6J–L were the dyed samples by I-KI_2,_ whose sieve tubes in phloem and xylem vessel were stained dark blue by I-KI_2_ solution (red arrow), the cells of the sponge tissue and palisade tissue also showed a darker blue color after staining. It can be seen that the accumulation of starch in the phloem of leaves with severe yellowing symptoms of Huanglong disease is more obvious.

### 3.3 Effects of antimicrobial lipopeptide on microflora in roots

#### 3.3.1 Flora diversity data analysis and species dilution curve

**Figure 6:**
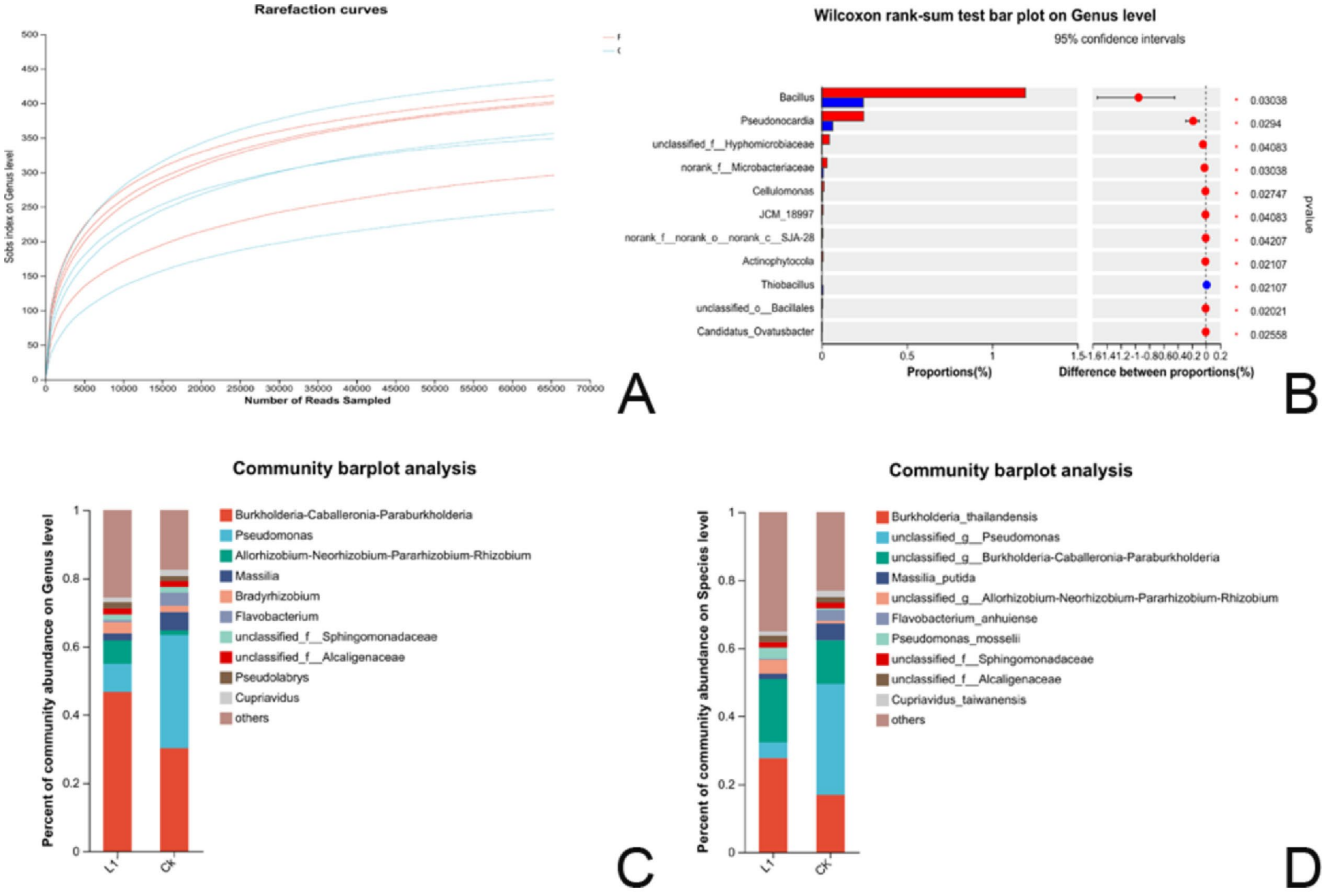
**(A–D)** Results of microflora analysis. **(A)** Dilution curve of the bacterial community sequencing library. The x-axis represents the amount of randomly extracted sequencing data, whereas the y-axis represents the number of observed species (Sobs index in OTUs). **(B)** The histogram of multispecies differences (genus) displays the taxonomic genus-level species names on the Y-axis, whereas the X-axis represents the average relative abundance of species across different groups. * 0.01 ≤ *p* ≤ 0.05, **(C)** Bar map of the community at the genus level; **(D)** Bar map of the community at the species level. The horizontal axis represents the group name, whereas the vertical axis denotes the proportion of species in the treatment group.

There were four biological replicates for both the L1 treatment group and the control group. According to the diversity data analysis. At the genus level, the five most common species were *Burkholderia-Caballeronia-Paraburkholderia*, *Pseudomonas, Allorhizobium-Neorhizobium-Pararhizobium-Rhizobium*, and *Masseille Massili A*. *Bradyrhizobium.* Sequence information and samples information statistics show in Supplementary Tables S7–S9. OTUs at a 97% similarity level were employed for the statistical analysis of bioinformation. The dilution curve trend of the eight samples indicated a gentle slope, with Good’s coverage reaching 99.91%. These features suggested that the percentage of microbial communities detected in both the treatment and control groups was near saturation, and the sequencing volume sufficiently covered most species in the samples (Figure 6A), OUT species classification table showed in Supplementary Table S10.

#### 3.3.2 Significance test of differences between groups

A significance test of differences between groups was also conducted to identify microbial biomarkers. A difference test histogram revealed significant variations in 11 genera at the genus level between the L1 treatment group and the control group (Wilcoxon rank-sum test, 0.01 ≤ *p* ≤ 0.05). The noteworthy genera included *Bacillus, Pseudonocardia*, *unclassified_f__Hyphomicrobiaceae*, *norank_f__Microbacteriaceae*, *Cellulomonas*, *JCM_18997*, *norank_f__norank_o__norank_c__SJA-28*, *Actinophytocola*, *Thiobacillus*, *unclassified_o__Bacillales*, *and Candidatus_Ovatusbacter* (Figure 6B and Supplementary Table S11).

#### 3.3.3 Community composition at the genus level

The top 10 species in terms of relative abundance at the genus level are visually represented as species column graphs. In the root systems, L1 and the control had abundances of *Burkholderia-Caballeronia-Paraburkholderia* (46.6 and 30.1%), *Pseudomonas* (8.3 and 33.2%), *Allorhizobium-Neorhizobium-Pararhizobium-Rhizobium* (6.8 and 1.3%), *Massilia* (2.0 and 5.3%), *Bradyrhizobium* (3.2 and 1.8%), *Flavobacterium* (1.6 and 3.8%), *unclassified_f__Sphingomonadaceae* (1.8 and 1.7%), *unclassified_f_Alcaligenaceae* (1.8 and 1.5%), *Pseudolabrys* (1.8 and 1.5%), *Cupriavidus* (1.3 and 1.9%), and others (25.6 and 17.4%). In the L1 treatment group, the abundances of *Burkholderia-Caballeronia-Paraburkholderia, Allorhizobium-Neorhizobium-Pararhizobium-Rhizobium,* and *Bradyrhizobium* were 1.55-fold, 5.23-fold, and 1.77-fold greater than those in the control group, respectively, suggesting that they were the dominant strains in the roots and closely associated with growth promotion and nitrogen fixation (Figure 6C and Supplementary Table S12).

#### 3.3.4 Community composition at the species level

On the basis of the results of species annotation, the top 10 species with the highest relative abundance at the species level were selected, and a species histogram was generated. In both the L1 and control samples, the relative abundances were as follows: *Burkholderia_thailandensis* (27.66 and 0.83%), *unclassified_g__Pseudomonas* (4.59 and 32.65%), *unclassified_g__Burkholderia-Caballeronia-Paraburkholderia* (18.66 and 12.82%), *Massilia_putida* (1.61 and 4.97%), *unclassified_g__Allorhizobium-Neorhizobium-Pararhizobium-Rhizobium* (4.03 and 0.69%), *Flavobacterium_anhuiense* (0.38 and 3.25%), *Pseudomonas_mosselii* (3.19 and 0.46%), *unclassified_f__Sphingomonadaceae* (1.68 and 1.74%), *unclassified_f__Alcaligenaceae* (1.83 and 1.56%), *Cupriavidus_taiwanensis* (1.33 and 1.93%), and others (35.00 and 23.05%). In the L1 treatment group, the abundances of *Burkholderia_thailandensis, unclassified_g__Burkholderia-Caballeronia-Paraburkholderia, unclassified_g__Allorhizobium-Neorhizobium-Pararhizobium-Rhizobium,* and *Pseudomonas_mosselii* were 1.64-fold, 1.46-fold, 5.84-fold, and 6.93-fold greater than those in the control group, respectively. These species appear to be the dominant strains in the root system (Figure 6D and Supplementary Table S13).

### 3.4 Analysis of nontarget metabolites in citrus plants

#### 3.4.1 Differentially abundant metabolites in leaves and roots (POS)

PLS-DA was used to assess the similarity between metabolites. According to the NEG and POS analysis and Q2 value scope Supplementary Figures S7A–D. The results indicated a clear differentiation and clustering of metabolites between the L1 group and the control group, irrespective of whether the sample was from the leaves or roots.

Following L1 treatment, 1,525 metabolites were identified by examining positive ion patterns. A comparison of the leaves and roots of the treated and control plants through positive ion analysis revealed that when the fold change >2 (*p* value <0.05), the metabolite differences reached statistical significance. When comparing L1 vs. CK, the main differentially abundant metabolites included (6/10) lipids and lipid-like molecules and (4/10) phenylpropanoids and polyketides.

When comparing R1 vs. RCK, the differential parent classes of the metabolites included (3/4) phenylpropanoids and polyketides and (1/4) organoheterocyclic compounds. The corresponding compounds consisted of (3/4) coumarins and their derivatives and (1/4) benzopyrans. Additionally, the corresponding compounds of class and Subclass were showed in Table 2 and Supplementary Tables S14, S15.

**Table 2:** Differentially abundant metabolites (POSs).

	Metabolite number	Fold change	*p*-value	Superclass	Class	Subclass	Scale
ID (L1 vs. L CK)	POS_25431	3.30	3.36E-07	Lipids and lipid-like molecules	Prenol lipids	Triterpenoids	1/10
	POS_26418	2.67	4.75E-06	Lipids and lipid-like molecules	Prenol lipids	Tetraterpenoidsl	2/10
	POS_23591	2.07	6.35E-04	Lipids and lipid-like molecules	Prenol lipids	Tetraterpenoids	
	POS_7398	2.52	1.79E-05	Lipids and lipid-like molecules	Fatty Acyls	Eicosanoids	1/10
	POS_7407	2.39	2.45E-05	Lipids and lipid-like molecules	Steroids and steroid derivatives	Androstane steroids	1/10
	POS_17077	4.70	1.61E-04	Lipids and lipid-like molecules	Prenol lipids	Terpene glycosides	1/10
	POS_24422	3.20	9.96E-04	Phenylpropanoids and polyketides	Isoflavonoids	Isoflavonoid C-glycosides	1/10
	POS_13848	2.60	1.01E-04	Phenylpropanoids and polyketides	Isoflavonoids	O-methylated isoflavonoids	3/10
	POS_15764	4.27	3.69E-06	Phenylpropanoids and polyketides	Flavonoids	O-methylated flavonoids	
	POS_9948	2.32	3.24E-04	Phenylpropanoids and polyketides	Flavonoids	O-methylated flavonoids	
ID R1 vs. RCK	POS_2653	3.06	1.01E-04	Phenylpropanoids and polyketides	Coumarins and derivatives	Furanocoumarins	2/4
	POS_8299	2.36	0.01	Phenylpropanoids and polyketides	Coumarins and derivatives	Furanocoumarins	
	POS_5932	2.12	1.85E-04	Organoheterocyclic compounds	Benzopyrans	1-benzopyrans	1/4
	POS_5935	4.59	0.023	Phenylpropanoids and polyketides	Coumarins and derivatives	Hydroxycoumarins	1/4

#### 3.4.2 Differentially abundant metabolites in leaves and roots (NEG)

After L1 treatment, the samples were assessed in negative ion mode (fold change >2, *p* value <0.05), revealing significant differences in metabolites. When comparing L1 vs. LCK, the classes of differentially abundant metabolites were composed of (6/8) phenylpropanoids and polyketides, (1/8) lipids and lipid-like molecules, and (1/8) organic acids and derivatives.

For the root system, the comparison between R1 and RCK revealed differential parental classes of metabolites, including (3/8) benzenoids, (4/8) phenylpropanoids and polyketides, and (1/8) lipids and lipid-like molecules. The corresponding compounds included (1/8) tetralins, (2/8) benzene and substituted derivatives, (4/8) cinnamic acids and their derivatives, and (1/8) fatty acyls. The corresponding class and subclass compounds are shown in Table 3 and Supplementary Tables S16, S17.

**Table 3:** Differentially abundant metabolites (NEGs).

	Metabolite number	Fold change	*p*-value	Superclass	Class	Subclass	Scale
ID (L1 vs. LCK)	NEG_15302	4.56	1.36E-07	Phenylpropanoids and polyketides	Flavonoids	Flavonoid glycosides	5/8
	NEG_38106	4.70	1.49E-06	Phenylpropanoids and polyketides	Flavonoids	Flavonoid glycosides	
	NEG_15294	2.54	1.01E-05	Phenylpropanoids and polyketides	Flavonoids	Flavonoid glycosides	
	NEG_27244	2.17	5.93E-05	Phenylpropanoids and polyketides	Flavonoids	Flavonoid glycosides	
	NEG_28035	2.78	8.29E-05	Phenylpropanoids and polyketides	Flavonoids	Flavonoid glycosides	
	NEG_20948	2.21	8.28E-07	Phenylpropanoids and polyketides	2-arylbenzofuran flavonoids	--	1/8
	NEG_8442	2.33	6.42E-05	Lipids and lipid-like molecules	Fatty Acyls	Fatty acids and conjugates	1/8
	NEG_2561	2.50	3.54E-03	Organic acids and derivatives	Hydroxy acids and derivatives	Medium-chain hydroxy acids and derivatives	1/8
ID (R1 vs. RCK)	NEG_6369	3.13	4.99E-06	Benzenoids	Tetralins	--	1/8
	NEG_1151	3.00	9.08E-04	Benzenoids	Benzene and substituted derivatives	Phenylpyruvic acid derivatives	1/8
	NEG_6495	3.90	0.02	Benzenoids	Benzene and substituted derivatives	Benzenesulfonic acids and derivatives	1/8
	NEG_9151	2.71	5.15 E-03.	Phenylpropanoids and polyketides	Cinnamic acids and derivatives	Hydroxycinnamic acids and derivatives	1/8
	NEG_4539	2.30	0.01	Phenylpropanoids and polyketides	Coumarins and derivatives	--	1/8
	NEG_13509	2.47	2.74 E-05	Phenylpropanoids and polyketides	Coumarins and derivatives	Furanocoumarins	1/8
	NEG_3015	2.54	0.31 E-03	Phenylpropanoids and polyketides	Coumarins and derivatives	Pyranocoumarins	1/8
	NEG_216	4.21	2.50 E-05	Lipids and lipid-like molecules	Fatty Acyls	Fatty acids and conjugates	1/8

As shown in Table 2 (POS), among the 24 subclasses of differentially abundant metabolites, 8 were modified by glycosylation. As shown in Table 3 (NEG), among the 29 subclasses of differentially abundant metabolites, 2 were altered by glycosylation, and 3 were modified by methylation.

#### 3.4.3 Effects of the L1 treatment on plant hormones

**Figure 7:**
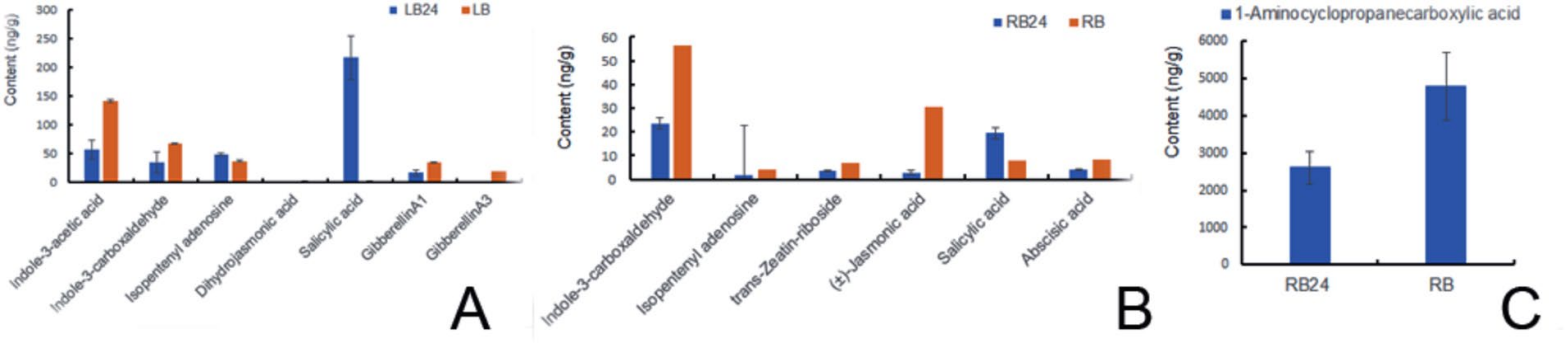
Hormone and SA changes induced by L1 treatment. **(A)** leaves, **(B,C)** roots.

After 24 h of spray-drenching with L1, the upregulation of SA in the leaves was very prominent, with the log_2_FC value reaching 6.53. The fold change values of the plant immunity hormones dihydrojasmonic acid and the cytokinin isopentenyl adenosine were increased 0.91- and 0.35-fold, respectively, which are closely associated with promoting growth and cell division. However, the log_2_FC values of IAA, *indole-3-carboxaldehyde*, GA1, and GA3 were downregulated by −1.31, −0.96, −0.98, and − 6.65 times, respectively (Figure 7A). After 24 h of treatment, the log_2_FC of SA in the roots increased 1.34-fold. Other hormones, such as *indole-3-carboxaldehyde*, isopentenyl adenosine, trans-zeatin riboside, 1-aminocyclopropanecarboxylic acid, (±)-jasmonic acid, abscisic acid, and gibberellin A1, were downregulated by −1.24, −1.43, −1.06, −0.80, −3.37, −1.03, and − 4.51 times, respectively (*p* < 0.05), Isopentenyl adenosine can induce cell division and proliferation and plays an important role in inducing bud differentiation, chloroplast development, nutrient transport, and anti-aging in plants. ACC is a precursor of naturally occurring ethylene biosynthesis, ACC log_2_FC in root was reduced by 0.80 times after L1 treatment (Figures 7B,C and Supplementary Tables S18–S21).

### 3.5 Phytohormone analysis of the L1 powder

The auxin contents of indole-3-acetic acid (IAA), 3-indolebutyric acid (IBA), *indole-3-carboxylic acid*, and *indole-3-carboxaldehyde* in the L1 complex were measured at 47.81, 0.72, 1.71, and 22.21 ng/g FW, respectively. The cytokinin contents including *N6-*isopentenyladenine, isopentenyl adenosine, and trans-zeatin-riboside, were found to be 12.46, 0.14, and 0.15 ng/g·FW, respectively. GA1, GA3, and GA7, with contents of 0.35, 0.02, and 0.01 ng/g FW, respectively. These phytohormones provide a solid basis for promoting plant growth and branching (Table 4). Also, the contents of (±)-jasmonic acid and salicylic acid were detected to be 0.32 and 3.62 ng/g·FW, respectively, indicating their may play additional plant immunity with systemic acquired resistance (SAR) for disease control. Content of ACC reached 105.72 ng/g.

**Table 4:** Determination of hormone content from the L1 complex with HPLC.

Name	L1_1	L1_2	L1_3	L1_4	Content (ng/g) DW	Content (ng/g) FW colonies^*^
Indole-3-acetic acid	460.0513527	430.7707792	531.9847143	489.6719502	478.1196991	47.81
3-Indolebutyric acid	7.260325653	5.358193939	10.91168846	5.195747313	7.18148884	0.72
Indole-3-carboxylic acid	13.51526148	15.99452387	18.20944106	20.81629226	17.13387967	1.71
Indole-3-carboxaldehyde	169.1365238	192.1197914	269.499507	257.7988903	222.1386781	22.21
N6-Isopentenyladenine	127.9838676	122.4088626	122.9462399	125.2849845	124.6559887	12.47
Isopentenyl adenosine	1.475923579	1.372689304	1.62093655	1.307242327	1.44419794	0.14
trans-Zeatin-riboside	1.516672545	1.516672545	1.516672545	1.516672545	1.516672545	0.15
1-Aminocyclopropanecarboxylic acid	1057.185494	1057.185494	1057.185494	1057.185494	1057.185494	105.72
Dihydrojasmonic acid	0.125417159	0.275161642	0.125417159	0.279071961	0.20126698	0.02
(±)-Jasmonic acid	2.328778579	4.272257937	2.04936457	3.991193754	3.16039871	0.31
Salicylic acid	25.20475284	29.78748218	44.01176365	45.30905324	36.07826298	3.61
Abscisic acid	3.474434577	3.978632832	3.729802218	3.815636133	3.74962644	0.37
GibberellinA1	3.505287849	3.505287849	3.505287849	3.505287849	3.505287849	0.35
GibberellinA3	0.196352476	0.196352476	0.196352476	0.196352476	0.196352476	0.02
GibberellinA7	0.101377645	0.101377645	0.20275529	0.101377645	0.126722056	0.01

*10 g of colonies can produce 1 gram of dry powder of L1.

### 3.6 Lipopeptides analysis of the L1 powder

The characteristic peak appeared in the retention time of Iturin A standard sample at 3.269 min. Under the same elution condition, the characteristic peak of L1 crude extract appeared at 2.325 min. The retention time is near the standard peak, and it is speculated that there are homologs of Iturin A in the crude extract (Supplementary Figures S8, S9).

The characteristic peak of Fengycin standard sample appeared at the retention time of 10.177 min. Under the same elution condition, the characteristic peak of L1 crude extract appeared at 10.000 min. The retention time is near the standard peak, and it is speculated that there are Fengycin homologs in L1 crude extract (Supplementary Figures S8, S9).

Four characteristic peaks appeared at the retention time of 12.062 min, 13.228 min, 13.974 min and 15.225 min for Surfact standard samples. Under the same elution condition, the characteristic peaks of L1 crude extract appeared at 12.129 min, 13.203 min, 14.021 min and 15.260 min. These peaks are near the standard peaks, indicating the presence of Surfact in the crude extract. Calculated by the standard curve equation, Iturin content was 13.95 ± 10.46 mg/kg, Fengycin content was 1.10 ± 0.91 mg/kg, Surfactins had 4 lipopeptide peaks with a total of 172.32 mg/kg, and the total of the three lipopeptides was 187.37 mg/kg (Supplementary Figures S8, S9).

## 4 Discussion and conclusion

In this study, the effectiveness of cyclic-lipopeptide and phytohormone complexes prepared from the fermentation of *Bacillus amyloliquefaciens strain* MG-2 in reducing *C*Las titers and stimulating host immune resistance were demonstrated after 105 days. Our findings revealed that L1 activated five defense enzymes, upregulated four host defense response-related genes and two hypersensitivity response genes, and increased the contents of SA, H2JA and IPA in leaves. This study also directly confirmed the increased SA content in roots after treated by L1 treatments. Analysis of the root flora indicated that the prevalent flora in the L1 treatment group potentially contributed to nitrogen fixation, antibacterial properties, and the optimization of the probiotic category of the roots and soil health. Furthermore, morphological observations demonstrated that L1 had a pronounced growth-promoting effect and increased plant viability, resulting in the transformation of yellow leaves into green leaves as well as high root mass and plant branching. Nontargeted metabolome analysis revealed upregulated differentially abundant metabolites in both L1 vs. CK and R1 vs. RCK. These results suggest that L1 can trigger host immune responses and enhance defense mechanisms, thereby improving root browning and starch blockage in phloem tissues. The application of the antibacterial CLPs and phytohormones of L1 coffers a novel strategy for curative and preventive combating HLB disease as well as promoting plant or root growth, underscoring its significant theoretical and practical implications.

### 4.1 Lipopeptides are important factors for resistance to HLB

Researchers have previously reported that Surfactins possess antiviral and antibacterial properties (Hassanisaadi, 2023; Huang et al., 2022; Zeriouh et al., 2014). The natural lipophilicity of surfactin enables it to integrate into cell membranes and disrupt the structure and function of pathogens, resulting in target death. In addition, surfactins with hydroxy fatty acid structure triggered plant immunity for long-lasting plant protection (Debois et al., 2015; Suchodolski et al., 2020; Hoff et al., 2021). HPLC analyses showed L1 powder contained 172.32 mg/kg surfactins, and some fengycin and iturin, with an average total concentration of 187.37 mg/kg (Table 5). The L1 powder formulation contained 50% of *Bacillus amyloliquefacins* fermentation solids. There is opportunity to develop high CLP metabolite fermentation such as *B. velezensis* UTB 96 or FZB42 (Vahidinasab et al., 2022).

**Table 5:** Concentration of lipopeptides in L1 with HPLC (mg/kg).

Lipopeptide	No. of samples	Retention time	Peak area	Concentration (mg/L)	Average concentration (mg/kg)
Iturin	S1	3.325	227,828	7.69	13.95 ± 10.46
	S2	3.352	1,025,832	31.5	
	S3	3.317	370,070	11.94	
	S4	3.315	125,211	4.64	
Fengycin	S2	10.117	41,112	2.68	1.10 ± 0.91
	S4	10.360	2,324	0.58	
	S5	10.277	1,077	0.51	
	S6	10.078	3,542	0.65	
Surfactin	S1 (peak 1)	13.203	252,688	57.72	44.16 ± 7.82
	S2 (peak 1)	13.208	158	39.64	
	S3 (peak 1)	13.150	182	39.64	
	S4 (peak 1)	13.175	176	39.64	
	S1 (peak 2)	14.021	99,964	24.04	22.41 ± 0.94
	S2 (peak 2)	14.019	1,137	21.88	
	S3 (peak 2)	14.100	1,279	21.88	
	S4 (peak 2)	14.087	228	21.86	
	S2 (peak 3)	14.825	2,378	46.66	46.85 ± 0.18
	S3 (peak 3)	14.783	4,774	47.03	
	S1 (peak 4)	15.260	15,582	59.7	58.90 ± 1.69
	S2 (peak 4)	15.295	6,959	57.58	
	S3 (peak 4)	15.403	21,940	61.27	
	S4 (peak 4)	15.439	4,820	57.05	
Total					187.37

### 4.2 Phytohormone analysis and plant and root growth effects

L1 powder with 50% fermentation solids contained high level of IAA, IBA, indole-3-caboxaldehyde auxins which are important for root growth and plant development. The IPA and t-zeatin riboside cytokinins are known plant growth regulator for cell division, photosynthesis and plant branching. They can prevent leave yellowing of HLB infection and improve citrus plant growth and fruit sizing. So far, the GA1, A3 and A7 contents are low in L1 powder. This may result less plant height effect as shown in earlier *Bacillus velezensis* study (Hu et al., 2021). Abscisic acid is important plant growth regulator and signaling molecule for abiotic stress tolerance such as drought and salinity (Tiwari et al., 2017), fruit ripening (Durán-Soria et al., 2020) and cereal grain filling grain (Ma et al., 2023). L1 powder only contained 3.47 mg/kg Abscisic acid. Further its fermentation yield improvement will be investigated for citrus plant abiotic stress tolerance and fruit yield and quality improvement.

It is not clear why L1 contained high levels of 1-aminocyclopropanecarbolic acid (ACC) and salicylic acid (SA) at 1057.18 and 36 ng/g, respectively. ACC may have negative effect on plant growth and senescence. SA is known as systemic acquired resistance (SAR) elicitor for plant defense. There is 3 ng/g Jasmonic acid present in L1. multiple reports have demonstrated that beneficial microorganisms activate SA and JA/ET signaling pathways in ISR, revealing the diversity and complexity of signaling pathways in ISR (Mamun et al., 2024; Samaras et al., 2021; Yuan et al., 2019). Those will provide additional mode of action against HLB disease.

### 4.3 L1 regulated expression of defense-related genes

NHO1 serves as a glycerol kinase that responds to various stimuli and plays a crucial role in plant innate immune responses, helping in resistance against different diseases. TaNHO1 can be activated by various plant hormones and is involved in wheat disease resistance (Xiao et al., 2022). Glutathione S-transferase (GST) is found in the cellular structures of various organisms. In plants, GSTs regulate growth, development, detoxification, and stress responses (Gullner et al., 2018; Otulak-Kozieł et al., 2022). HSP90 acts as a molecular chaperone, assisting in protein folding and degradation and regulating various physiological processes, including apoptosis, the cell cycle, and cell signal transduction (Ohama et al., 2016). Previous research has highlighted the significant role of the transcription factor WRKY in pathogen-induced defense mechanisms (Eulgem et al., 2000). In the case of bananas, MaWRKY1 and MaWRKY2 contribute to resistance against banana anthracnose induced by SA and methyl jasmonate (MeJA) by binding to the promoters of disease-related genes (Shan et al., 2016). In our study, treatment with L1 resulted in significantly increased the relative expression levels of defense-related genes (*nho1, WRKY22, WRKY24, and WRKY33*) and hypersensitivity response genes (*GST1 and HSP90*).

### 4.4 L1 changed the abundance of dominant flora

Community composition analysis revealed four dominant bacterial species in the roots following L1 treatment: *Burkholderia_thailandensis*, *unclassified_g__Burkholderia-Caballeronia-Paraburkholderia*, *unclassified_g__Allorhizobium-Neorhizobium-Pararhizobium-Rhizobium*, and *Pseudomonas_mosselii*. These bacteria are closely associated with bolstering antibacterial defenses, maintaining ecological equilibrium, and promoting nitrogen fixation. Previous studies have shown that *Burkholderia thailandensis* E264 was initially isolated from rice fields in Thailand.

Thailandepin A is a type of histone deacetylase inhibitor (HDACI) that bears structural similarity to the anticancer drug FK228. Owing to their antifouling and anticorrosion properties, long-chain rhamnolipids derived from bacteria have significant potential as biosurfactants for industrial water systems. These lipids have been demonstrated to inhibit biofilm formation by 50–90% (Jimoh et al., 2023). In the lower field region, *Burkholderia-Caballeronia-Paraburkholderia* was identified as the predominant and indicative bacterium in the 20–40 cm soil layer within the direct interaction zone between corn and peanut crops. Previous studies have established a strong association between this bacterium and the mitigation of challenges related to continuous cropping practices (Li H. et al., 2022). *Allorhizobium-Neorhizobium-Pararhizobium-*Rhizobium has been found to stimulate the growth of microalgae, primarily through the utilization of ammonia nitrogen. These bacteria increase the nitrification of ammonia nitrogen and consume a significant amount of oxygen in the system for their own growth (Idowu et al., 2024). The natural pyrazolotriazine pseudoiodinine from *Pseudomonas mosselii* 923 inhibits plant bacterial and fungal pathogens (Yang et al., 2023). *Pseudomonas* mosieri BS011 has been shown to have an anti-Fusarium oryzae effect. Furthermore, *Pseudomonas mosselii* (CCTCC NOM2018534) has antibacterial properties against rice stalk disease, rice blast disease, malignant seedling disease, and white leaf blight. These reports provide reference and enlightenment for us to further study the dominant bacteria caused by L1 treatment and explain the role of related bacteria.

### 4.5 L1 treatment caused the change of metabolites

Secondary metabolism plays crucial roles in the survival and adaptability of plants within their environment. Moreover, the diverse range of secondary metabolites serves as an inherent driving force for enhancing plant disease resistance. Under natural conditions, plants produce numerous secondary metabolites. These metabolites can interact with beneficial microbes, modulate plant growth and immune processes, and inhibit the growth or metabolism of pathogenic microorganisms. Beneficial microbes can be attracted by root exudates, which establish a distinct community of rhizosphere microorganisms and enhance the biofilm formation of beneficial microbes (Zhang et al., 2015). Consequently, secondary metabolites secreted by these beneficial microorganisms can directly counteract pathogenic bacteria and act as immune elicitors to induce ISR (Prsic and Ongena, 2020). Phenazines produced by beneficial *Pseudomonas* bacteria exhibit antifungal activity and can elicit ISR (Chin-A-Woeng et al., 2003). These compounds often possess distinct functionalities. Previous studies have shown that plant triterpenoids, which are natural compounds consisting of isoprene units. Terpenes are the largest among plant secondary metabolites PSMs and have been extensively studied for their potential as antimicrobial, insecticidal, and weed control agents (Ninkuu et al., 2021). Coumarins, a group of phenylpropanolactones with benzopyranone nuclei, are secondary metabolites derived from the shikimic acid pathway through phenylalanine or tyrosine (Huang et al., 2024). One specific coumarin, 7-hydroxycoumarin, has antibacterial properties (Sebastian et al., 2013) and can enhance the water solubility and bacteriostatic properties of chitosan when modified through amidation. Secondary metabolites of coumarins and lignin extracted from *Artemisia annua* have also been shown to effectively inhibit *Fusarium oxysporum* (Li K. et al., 2019). Arachidonic acids play a vital role in the insect-mediated elimination of bacteria, fungi, parasites, and viruses (Stanley-Samuelson et al., 1991). In this study, some mentioned secondary metabolites were significantly upregulated after treatment with L1 (fold change >2, *p* < 0.05), indicating their potential role in sterilizing *C*las or inducing immune resistance. Because the pathogen can not be pure culture, the verification of the antibacterial activity of metabolites has limitations.

In conclusion, the cyclic-lipopeptide and phytohormone complex powder L1 prepared from the batch fermentation of *Bacillus amyloliquefaciens* MG-2 has reduced the *C*Las titer, promoted plant growth, increased root mass, induced host immune responses, enhanced beneficial bacteria levels and soil health, and increased the content of phytohormones in leaves and roots. This study presents innovative and multiple mode of actions of Bacillus PGPB to manage HLB disease and to promote citrus plant or root growth, which represents a significant advancement in this field and warrants further exploration.

**Figure 8:**
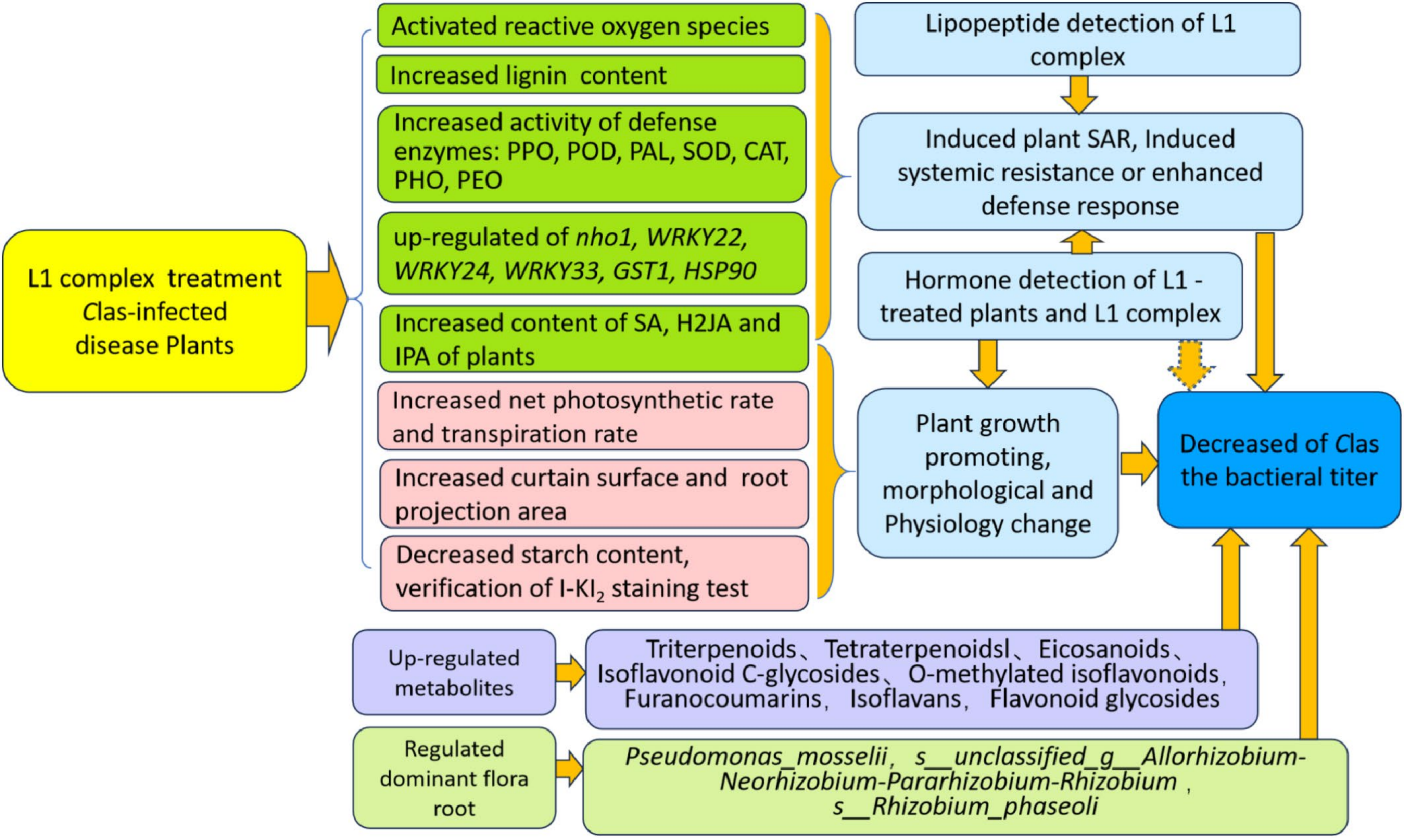
Diagram of the multiple functions of the lipopeptide and phytohormone complex L1.

This research focused on examining the effects of L1 treatment on affected plants from various perspectives (Figure 8). Although complete eradication of *C*Las in infected plants has not been achieved yet. Further improvement of high cell mass fermentation for producing more CLP and phytohormone metabolites and whole plant irrigation treatment methods will be investigated for total biological effectiveness of endophytic Bacillus PGPB to combat HLB disease and to improve citrus productivity with yield increase and fruit quality.

## Data Availability

The datasets presented in this study can be found in online repositories. The names of the repository/repositories and accession number(s) can be found in the article/Supplementary material.

## Supplementary material

SUPPLEMENTARY FIGURE S1 **(A-D)** Construct of plasmid vector of pEZclone-A04-1 and pEZclone-COX -1 and sequential analysis.

SUPPLEMENTARY FIGURE S2 **(A-F)** Standard curve of target gene and house keep gene for qPCR.

SUPPLEMENTARY FIGURE S3 **(A-D)** Average maximum and minimum temperatures from June to September of 2022.

SUPPLEMENTARY FIGURE S4 **(A-B)** Gel electrophoresis of PCR amplify product.

SUPPLEMENTARY FIGURE S5 Counting process for two percentage comparison hypothesis tests.

SUPPLEMENTARY FIGURE S6 **(A-L)** I-KI2 staining of transverse section of leaves vein.

SUPPLEMENTARY FIGURE S7 **(A-D)** Partial least squares method (PLS-DA) analysis of metabolites.

SUPPLEMENTARY FIGURE S8 Standard curve for the determination of three lipopeptides.

SUPPLEMENTARY FIGURE S9 XIC diagram of hormone standard and tested samples.
