# Supplementary material for: Investigation of the anti-Huanglongbing effects using antimicrobial lipopeptide and phytohormone complex powder prepared from Bacillus amyloliquefaciens MG-2 fermentation
Source: Front Microbiol. 2024 Dec 18;15:1458051. doi: 10.3389/fmicb.2024.1458051 (PMC11694225; doi:10.3389/fmicb.2024.1458051)
Supplement: SUPPLEMENTARY FIGURE S1 — (A-D) Construct of plasmid vector of pEZclone-A04-1 and pEZclone-COX -1 and sequential analysis. [file Data_Sheet_1.zip › Table S1-21/Table S18 S19 Plant hormone.pdf]

Table S18 Plant hormone mass spectrometry detection parameters

NO.	Component Name	Q1	Q3	RT[min]	DP	CE
1	Indole-3-acetic acid	176	130	4.56	50	20
2	3-Indolebutyric acid	204	186	5.07	87	17
3	Indole-3-carboxylic acid	160	116	4.38	-38	-21
4	Methyl indole-3-acetate	190	130	5.42	64	19
5	Indole-3-carboxaldehyde	146	118	4.53	80	20
6	N6-Isopentenyladenine	204	136	3.99	70	20
7	Isopentenyl adenosine	336	204	4.21	85	23
8	trans-Zeatin-riboside	352	220	3.54	95	27
9	trans-Zeatin	220	136	2.54	70	25
10	Dihydrozeatin	222	136	2.71	70	29
11	Kinetin	216	81	3.81	115	23
12	Methylsalicylate	151	91	6.07	-50	-30
13	Brassinolide	481.7	445.5	6.02	80	15
14	Methyl jasmonate	225	151	6.46	75	15
15	Dihydrojasmonic acid	211	59	5.46	-85	-15
16	N-Jasimonic acid-isoleucine	324	278	5.47	85	17
17	(±)-Jasmonic acid	209	59	5.16	-86	-17
18	Salicylic acid	137	93	4.71	-70	-23
19	Abscisic acid	263	153	4.72	-85	-16
20	GibberellinA1	347	273	4.12	-140	-32
21	GibberellinA3	345	143	4.1	-110	-32
22	GibberellinA4	331	313	5.39	-95	-24
23	GibberellinA7	329	223	5.31	-90	-26
24	1-Aminocyclopropanecarboxylic acid	102	56	1.09	77	20
25	Indole-3-acetic acid-D4	180	133	4.56	40	31
26	Jasmonic Acid-D5	214	62	5.16	-60	-15
27	N6-Isopentenyladenine-D6	210.2	137.2	3.99	40	21
28	Dihydrozeatin-D3	225	136.2	2.71	80	29
29	GibberellinA1-D4	351	307	4.12	-49	-29
30	Salicylic acid-D4	141	97	4.71	-50	-23
31	Abscisic acid-D6	269	159	4.72	-50	-24

Table S19 Regression Equation for hormone content determination

NO.	Phytohormone	Regression Equation	R^2
1	Indole-3-acetic acid	y = 0.06114 x + 0.00537	0.99376
2	3-Indolebutyric acid	y = 0.10849 x + -9.05807e^{-4}	0.99633
3	Indole-3-carboxylic acid	y = 0.02064 x + 0.00586	0.99853
4	Methyl indole-3-acetate	y = 0.07358 x + 0.00686	0.99746
5	Indole-3-carboxaldehyde	y = 0.06644 x + 0.06543	0.99705
6	N6-Isopentenyladenine	y = 0.16470 x + 0.02251	0.99480
7	Isopentenyl adenosine	y = 0.14982 x + 0.01338	0.99524
8	trans-Zeatin-riboside	y = 0.50666 x + 0.04507	0.99409
9	trans-Zeatin	y = 0.22013 x + -0.16326	0.99079
10	Dihydrozeatin	y = 0.38467 x + 0.14718	0.99507
11	Kinetin	y = 0.01650 x + 8.63929e^{-4}	0.99787
12	Brassinolide	y = 0.00420 x + 0.02579	0.99634
13	Methyl jasmonate	y = 3.36326 x + 0.05983	0.99536
14	N-Jasimonic acid isoleucine	y = 2.91545 x + -0.22875	0.99450
15	1-Aminocyclopropanecarboxylic acid	y = 0.00108 x + 0.09386	0.99335
16	Methylsalicylate	y = 6.42094 x + 4850.58949	0.99143
17	Dihydrojasmonic acid	y = 1.89226 x + -0.01116	0.99896
18	(±)-Jasmonic acid	y = 1.32842 x + 0.20784	0.99898
19	Salicylic acid	y = 0.17659 x + 0.07026	0.99600
20	Abscisic acid	y = 0.02186 x + 0.00248	0.99524
21	GibberellinA1	y = 0.82556 x + 0.05752	0.99624
22	GibberellinA3	y = 0.01485 x + 0.00184	0.99150
23	GibberellinA4	y = 0.00452 x + 0.00230	0.99585
24	GibberellinA7	y = 0.03361 x + 0.01040	0.99401
