# Supplementary material for: Investigation of the anti-Huanglongbing effects using antimicrobial lipopeptide and phytohormone complex powder prepared from Bacillus amyloliquefaciens MG-2 fermentation
Source: Front Microbiol. 2024 Dec 18;15:1458051. doi: 10.3389/fmicb.2024.1458051 (PMC11694225; doi:10.3389/fmicb.2024.1458051)
Supplement: SUPPLEMENTARY FIGURE S1 — (A-D) Construct of plasmid vector of pEZclone-A04-1 and pEZclone-COX -1 and sequential analysis. [file Data_Sheet_1.zip › Table S1-21/Table S3 S4 S5 S6 Raw data of QPCR and Percentage reduction in titer.pdf]

Table S3 The raw data and statistic analysis of qPCR detection about citrus Huanglongbing disease before L1 fermentation complex treatment

Before treatment	A04	COX	Δt	Ct average	Variance	ΔΔt	Error of ΔΔt	$2^{-ΔΔt}$	Error of $2^{-ΔΔt}$	Error
-	33.58	15.21	18.37							
	33.61	15.54	18.08							
	33.27	15.03	18.24	18.23	0.15	0.00	0.15	1.00	1.11	0.11
+	29.62	13.93	15.69							
	29.83	14.13	15.70							
	29.37	13.90	15.47	15.62	0.13	2.61	2.74	6.10	6.68	0.58
CK C4	31.00	14.70	16.29							
	30.44	14.34	16.11							
	30.91	14.59	16.32	16.24	0.12	1.99	2.11	3.98	4.31	0.33
CK C15	30.05	12.96	17.09							
	30.11	12.98	17.13							
	30.56	13.28	17.29	17.17	0.10	1.06	1.16	2.09	2.24	0.16
CK C17	31.70	13.96	17.75							
	31.09	14.13	16.96							
	30.94	13.45	17.49	17.40	0.40	0.83	1.23	1.78	2.35	0.57
CK C48	31.54	14.19	17.36							
	31.60	14.64	16.96							
	31.45	14.28	17.17	17.16	0.20	1.07	1.27	2.10	2.41	0.31
CK C70	31.79	15.08	16.71							
	31.95	15.54	16.42							
	31.99	15.06	16.94	16.69	0.26	1.54	1.80	2.91	3.49	0.58
CK C97	30.08	14.31	15.77							
	30.43	14.78	15.65							
	30.82	14.95	15.87	15.76	0.11	2.47	2.57	5.52	5.96	0.43
C24	31.48	14.73	16.75							
	31.09	14.61	16.48							
	31.63	14.82	16.81	16.68	0.18	1.55	1.72	2.92	3.30	0.38
C32	30.53	14.04	16.49							
	30.22	13.75	16.47							
	30.51	13.83	16.68	16.55	0.11	1.68	1.80	3.21	3.47	0.26
C46	31.94	15.41	16.53							
	32.04	14.90	17.14							
	31.83	15.40	16.43	16.70	0.38	1.53	1.91	2.89	3.76	0.87
C51	32.94	15.56	17.37							
	32.59	15.47	17.13							
	32.15	15.10	17.05	17.18	0.17	1.05	1.21	2.07	2.32	0.25
C76	31.68	14.97	16.71							
	31.16	14.54	16.63							
	31.47	14.45	17.01	16.78	0.20	1.45	1.65	2.73	3.14	0.41
C90	29.23	13.16	16.08							
	29.83	13.99	15.84							
	29.61	13.34	16.26	16.06	0.21	2.17	2.38	4.50	5.20	0.71

Table S4 The raw data and statistic analysis of qPCR detection about citrus Huanglongbing disease after L1 Fermentation complex treatment 15 times

After treatment	A04	COX	Δt	Ct average	Variance	ΔΔt	Error of ΔΔt	$2^{-ΔΔt}$	Error of $2^{-ΔΔt}$	Error
-	33.25	15.44	17.81							
	33.71	15.77	17.95							
	33.37	15.27	18.10	17.95	0.15	0.00	0.15	1.00	1.11	0.11
+	29.72	13.79	15.93							
	29.93	13.98	15.95							
	29.04	13.75	15.29	15.72	0.38	2.23	2.61	4.69	6.09	1.40
CK C4	31.38	14.45	16.93							
	30.91	14.13	16.79							
	31.26	14.37	16.88	16.87	0.07	1.09	1.16	2.12	2.23	0.11
CK C15	31.04	12.83	18.21							
	30.85	12.85	18.00							
	31.45	12.83	18.62	18.28	0.31	0.32	0.01	0.80	0.99	0.19
CK C17	31.85	14.49	17.35							
	31.43	14.02	17.41							
	31.67	14.03	17.64	17.47	0.15	0.49	0.64	1.40	1.55	0.15
CK C48	31.73	14.81	16.91							
	31.97	14.35	17.62							
	32.44	14.02	18.42	17.65	0.76	0.30	1.06	1.23	2.08	0.85
CK C70	31.86	14.02	17.83							
	31.92	14.27	17.65							
	31.64	14.69	16.95	17.48	0.47	0.48	0.94	1.39	1.92	0.53
CK C97	31.00	13.91	17.09							
	30.79	14.45	16.34							
	31.19	13.79	17.39	16.94	0.54	1.01	1.55	2.02	2.93	0.92
C24	32.42	14.36	18.06							
	32.54	14.51	18.04							
	32.52	14.34	18.18	18.09	0.08	0.14	0.06	0.91	0.96	0.05
C32	31.06	13.94	17.11							
	30.75	13.59	17.16							
	31.45	13.65	17.80	17.36	0.39	0.60	0.98	1.51	1.98	0.46
C46	32.54	14.16	18.38							
	32.38	14.49	17.89							
	32.33	14.08	18.26	18.18	0.25	0.22	0.03	0.86	1.02	0.17
C51	32.65	15.08	17.57							
	32.14	15.30	16.85							
	32.10	14.92	17.18	17.20	0.36	0.76	1.12	1.69	2.17	0.48
C76	32.67	14.71	17.96							
	31.60	14.37	17.23							
	32.16	14.02	18.14	17.78	0.48	0.18	0.66	1.13	1.58	0.45
C90	32.03	13.99	18.04							
	32.49	13.65	18.84							
	32.23	13.43	18.80	18.56	0.46	0.61	0.15	0.66	0.90	0.24

Table S5 The raw data and statistic analysis of qPCR detection about citrus Huanglongbing disease after L1 Fermentation complex treatment 18 times

After treatment	A04	COX	Δt	Ct average	Variance	ΔΔt	Error of ΔΔt	$2^{-ΔΔt}$	Error of $2^{-ΔΔt}$	Error
-	33.41	15.36	18.05							
	33.84	15.70	18.14							
	33.40	15.22	18.18	18.12	0.07	0.00	0.07	1.00	1.05	0.05
+	29.87	13.76	16.11							
	29.96	13.92	16.04							
	29.18	13.51	15.67	15.94	0.24	2.19	2.42	4.55	5.37	0.81
C4	31.49	14.37	17.12							
	30.95	14.05	16.90							
	31.30	14.30	16.99	17.00	0.11	1.12	1.23	2.18	2.35	0.17
C15	31.19	12.76	18.43							
	31.02	12.83	18.19							
	31.65	12.74	18.91	18.51	0.37	0.38	0.02	0.77	0.99	0.22
C17	32.04	14.47	17.57							
	31.63	13.97	17.65							
	31.85	14.00	17.85	17.69	0.14	0.43	0.58	1.35	1.49	0.14
C48	31.99	14.75	17.24							
	32.15	14.33	17.82							
	32.50	13.99	18.52	17.86	0.64	0.27	0.91	1.20	1.87	0.67
C70	31.69	13.99	17.70							
	32.07	14.19	17.88							
	31.80	14.62	17.18	17.59	0.37	0.54	0.90	1.45	1.87	0.42
C97	31.07	13.90	17.17							
	30.99	14.35	16.64							
	31.02	13.74	17.28	17.03	0.34	1.10	1.44	2.14	2.71	0.58
C24	32.45	14.29	18.16							
	32.65	14.47	18.19							
	32.59	14.25	18.34	18.23	0.10	0.10	0.01	0.93	1.00	0.06
C32	32.51	13.88	18.63							
	31.86	13.51	18.36							
	31.55	13.57	17.97	18.32	0.33	0.19	0.13	0.87	1.10	0.22
C46	32.50	14.06	18.44							
	32.50	14.46	18.04							
	32.26	14.01	18.25	18.24	0.20	0.12	0.08	0.92	1.06	0.13
C51	32.49	14.60	17.89							
	32.29	14.20	18.09							
	32.18	14.82	17.36	17.78	0.38	0.34	0.72	1.27	1.65	0.38
C76	32.79	14.46	18.33							
	32.80	14.19	18.61							
	32.27	13.99	18.28	18.41	0.18	0.28	0.11	0.82	0.93	0.11
C90	32.30	13.97	18.33							
	32.97	13.57	19.40							

	**32.57**	**13.35**	**19.21**	**18.98**	**0.57**	**0.86**	**0.29**	**0.55**	**0.82**	**0.27**

Table S6 Percentage reduction in cells/gram tissues

	No.	Before treatment, cells/gram tissues		After treatment 15 times, cells/gram tissues		Percentage reduction in titer (cells/gram tissues) （%）	After treatment 18 times, cells/gram tissues		Percentage reduction in titer (cells/gram tissues) （%）
CK	C4	2818.4	2587.3	2187.8	1865.7	27.9A*	2108.1	1759.7	31.9%A*
	C15	3981.1		2290.9			2053.9		
	C17	2089.3		1584.9			1425.9		
	C48	1737.8		1230.3			1120.4		
	C70	1349.0		1445.4			1416.6		
	C97	3548.1		2454.7			243.3		
L1	C24	1862.1	2621.7	933.2	1258.6	51.9B	891.9	1122.7	57.2%B
	C32	3630.7		2344.2			1310.0		
	C46	1318.2		954.9			977.1		
	C51	891.2		1047.8			1042.9		
	C76	1862.1		1174.9			857.7		
	C90	6166.0		1096.5			1656.4		

$P<0.01$ $\alpha=2.575$
